# Supplementary material for: Galloylated liposomes enable targeted drug delivery by overcoming protein corona shielding
Source: Nat Commun. 2025 Aug 25;16:7926. doi: 10.1038/s41467-025-63198-4 (PMC12378351; doi:10.1038/s41467-025-63198-4)
Supplement: Supplementary file 1 — Supplementary Information [file 41467_2025_63198_MOESM1_ESM.pdf]

Supplementary Information

Galloylated Liposomes Enable Targeted Drug Delivery by Overcoming Protein Corona Shielding

Jinbo Li[1#], Jiang Yu[1#], Jia Song[1], Yingxi Zhang[1], Ning Li[1], Zhaomeng Wang[2], Meng Qin[1], Mingming Zhao[1], Baoyue Zhang[1], Ruiping Huang[1], Shuang Zhou[1], Yubo Liu, Zhonggui He[1], Hongzhuo Liu[1,3,] *, Dan Liu[4,] *, Yongjun Wang[1,3,] *.

[1] Wuya College of Innovation, Shenyang Pharmaceutical University, Shenyang 110016, China

[2] Department of Oncology, Cancer Stem Cell and Translational Medicine Lab, Innovative Cancer Drug Research and Development Engineering Center of Liaoning Province, Shengjing Hospital of China Medical University, Shenyang 110004, China.

[3] Joint International Research Laboratory of Intelligent Drug Delivery Systems, Ministry of Education, Shenyang Pharmaceutical University, Shenyang 110016, China

[4] Key Laboratory of Structure-Based Drugs Design & Discovery of Ministry of Education, Shenyang Pharmaceutical University, Shenyang, 110016, China

* Corresponding author E-mail addresses: liuhongzhuo@syphu.edu.cn (H. Liu), liudan@syphu.edu.cn (D. Liu), wangyongjun@syphu.edu.cn (Y. Wang).

[#] Both authors contributed equally.

The supplementary information includes:

Supplementary Table. 1-7

Supplementary Fig. 1-59

Supplementary Table 1 The influence of hydrophobic tails of GA-fatty acid lipids on drug loading capacity and protein absorption efficiency. (with 0.025 mol% Trf) (mean value ± sd, *n* = 3).

**Lipid composition （mol ratio）**	**Blank liposome**			**DOX-loaded liposome**			
	Size (nm)	PDI	Binding rate of TRF-RB （%）	Size (nm)	PDI	Binding rate of Trf-RB （%）	Encapsulated efficiency （%）
HSPC: Chol=60:40	125.6±3.72	0.156±0.043	6.98±3.03	121.3±0.866	0.155±0.047	3.72±0.46	98.41±1.77
HSPC: Chol: GA-lipid18(1)=60:30:10	147.2±4.20	0.169±0.053	70.57±6.04	199.0±5.029	0.147±0.054	77.32±1.17	96.57±0.92
HSPC: Chol: GA-lipid18(0)=60:30:10	——	——	——	——	——	——	——
HSPC: Chol: GA-lipid16(0)=60:30:10	116.5±1.41	0.128±0.030	69.33±5.43	162.2±0.404	0.193±0.054	53.74±0.12	81.26±0.56
HSPC: Chol: GA-lipid14(0)=60:30:10	100.2±3.82	0.124±0.008	55.20±7.91	181.9±5.805	0.234±0.063	64.77±1.21	85.42±2.64
HSPC: Chol: GA-lipid12(0)=60:30:10	120.6±1.72	0.121±0.018	68.80±11.35	302.8±2.857	0.172±0.033	37.26±27.08	67.04±0.05

Supplementary Table 2 The influence of lipid composition on DOX encapsulated efficiency and TRF absorption efficiency. (with 0.025 mol% Trf) (mean value ± sd, *n* = 3).

**Lipid composition (mol ratio)**	**Dox: lipid (mass ratio)**	**Encapsulated efficiency（%）**	**Binding rate of Trf-RB （%）**
HSPC: Chol=80:20	1:10	95.8±5.78	2.214±2.15
HSPC: Chol: GA-lipid18(1) = 80:20:10		52.69±8.98	25.08±0.31
HSPC: Chol=60:40		98.96±2.33	11.83±4.28
HSPC: Chol: GA-lipid18(1) = 60:30:10		99.15±2.86	72.81±5.41
HSPC: Chol: GA-lipid18(1) = 60:30:4.5		93.20±7.69	39.27±13.68

The ratio of chol in GA-lipid can influence the drug loading efficiency of DOX, 30% Chol is better. The content of GA-lipid is 8.9, which is the maximum addition amount for stable liposome with best DOX loading efficiency and Trf absorption efficiency.

Supplementary Table 3 The influence of binding linker of GA-Chol on DOX loading capacity and Trf absorption efficiency. (with 0.025 mol% Trf) (mean value ± sd, *n* = 3).

**Lipid composition （mol ratio）**	**Size (nm)**	**PDI**	**Zeta potential (mV)**	**DOX (EE%)**	**Size (nm)**	**PDI**	**Zeta potential (mV)**	**Binding rate of TRF-RB （%）**
HSPC: Chol=60:40	128.7±2.417	0.133±0.053	-15.0±2.33	97.11±1.75	126.3±1.464	0.186±0.008	-15.1±3.08	19.39±9.64
HSPC: Chol: Chol-P0-GA=60:30:10	132.4±3.430	0.197±0.029	-17.1±1.22	97.91±1.33	134.0±0.513	0.145±0.043	-15.5±0.55	66.97±10.71
HSPC: Chol: Chol-P1-GA=60:30:10	105.6±1.504	0.171±0.032	-17.3±0.95	98.70±1.84	104.4±1.950	0.116±0.051	-17.4±0.80	68.45±8.53
HSPC: Chol: Chol-P3-GA=60:30:10	122.7±5.497	0.200±0.056	-15.7±0.75	95.65±1.13	128.6±6.218	0.356±0.051	-13.2±0.46	67.63±5.08
HSPC: Chol: Chol-P5-GA=60:30:10	166.2±6.213	0.237±0.023	-15.9±0.32	89.99±2.10	193.2±5.415	0.286±0.017	-13.6±0.49	74.28±3.97

Supplementary Table 4 Pharmacokinetic parameters of DOX solution and various liposomal formulations after intravenous administration (*n* = 3).

	Cmax (ug/L)	AUC(0-t) (ug/L*h)	t1/2z (h)	CLz (L/h/kg)	Vz (L/kg)
**DOX**	2.09±0.69	0.69±0.13	0.53±0.25	3.552±0.556	2.701±1.348
**DOX-lipo**	105.50±4.67	1643.94±160.14	15.51±1.72	0.001±0.001	0.033±0.006
**Trf/DOX-lipo**	98.09±1.90	1573.78±36.03	12.19±1.49	0.002±0.001	0.028±0.004
**GA-P0-DOX-lipo**	186.99±7.15	1081.09±138.31	4.63±0.093	0.002±0.001	0.016±0.005
**Trf@GA-P0-DOX-lipo**	230.18±15.18	1985.25±63.52	7.64±1.37	0.001±0.001	0.014±0.003
**GA-P1-DOX-lipo**	240.53±22.46	2153.23±207.12	7.69±2.48	0.001±0.001	0.013±0.004
**Trf@GA-P1-DOX-lipo**	271.40±39.28	2659.80±315.77	11.48±1.11	0.001±0.001	0.015±0.002
**GA-P3-DOX-lipo**	20.99±3.20	22.41±4.86	1.03±0.62	0.106±0.033	0.141±0.066
**Trf@GA-P3-DOX-lipo**	25.71±2.10	54.34±13.30	1.77±1.20	0.048±0.012	0.109±0.048

Supplementary Table 5 The formulation of DXdd-loaded liposomes (*n* = 3).

Lipid composition （mole ratio）	Size (nm)	PDI	Zeta potential (mV)	DXdd (EE%)	Size (nm)	PDI	Zeta potential (mV)	Binding rate of TRA-FITC （%）
HSPC: Chol=60:40	128.7±2.417	0.133±0.053	-15.0±2.33	92.45±0.66	128.4±2.136	0.164±0.036	-18.6±0.306	9.74±1.05
HSPC: Chol: GA-P0-Chol =60:30:10	132.4±3.430	0.197±0.029	-17.1±1.22	95.18±6.74	134.0±2.230	0.168±0.019	-14.6±2.21	45.23±6.97
HSPC: Chol: GA-P1-Chol=60:30:10	105.6±1.504	0.171±0.032	-17.3±0.95	96.28±7.31	102.6±1.943	0.130±0.080	-13.0±1.93	70.3±2.43
HSPC: Chol: GA-P3-Chol=60:30:10	122.7±5.497	0.200±0.056	-15.7±0.75	77.34±6.90	123.7±3.075	0.320±0.022	-13.3±0.551	40.00±0.66
HSPC: Chol: GA-P5-Chol=60:30:10	166.2±6.213	0.237±0.023	-15.9±0.32	68.30±2.27	175.2±3.535	0.221±0.077	-13.5±0.702	32.83±7.03

Supplementary Table 6 ITC-derived thermodynamic parameters for the binding of trastuzumab to GA-lipo.

Titrant	Titrand	KD(M)	N	△H(KJ/mol)	△S(J/mol·K)
GA-lipo	trastuzumab	3.29e-07	1.83	-38.55	-5.25

Supplementary Table 7 The characterization of different antibody-adsorbed GA-p1-lipo. (mean value ± sd, *n* = 3).

	Parameter	Batch 1	Batch 2	Batch 3	Mean ± SD	CV%
TRA@GA-P1-lipo	Adsorption efficiency (%)	78.47±1.06 (CV=1.35)	66.91±1.89 (CV=1.89)	64.10±5.27 (CV=5.62)	69.82±7.18	10.29
	Size (nm)	103.2±1.71 (CV=1.66)	104.9±5.25 (CV=5.01)	104.9±0.35 (CV=0.33)	104.4±2.89	2.77
	PDI	0.043±0.040	0.051±0.023	0.046±0.025	0.047±0.026	
	Zeta potential (mV)	-14.03±0.94	-13.9±0.87	-13.96±0.66	-13.97±0.73	
Cet@GA-P1-lipo	Adsorption efficiency (%)	66.15±2.56 (CV=3.87)	58.31±3.76 (CV=6.48)	76.14±2.24 (CV=2.95)	66.87±8.14	12.1
	Size (nm)	106.0±1.90 (CV=1.79)	101.56±1.37 (CV=1.37)	104.3±3.81 (CV=3.66)	103.9±2.97	2.86
	PDI	0.060±0.039	0.111±0.062	0.033±0/018	0.068±0.050	
	Zeta potential (mV)	-8.23±1.46	-8.97±0.524	-8.4±0.346	-8.55±0.86	
Nimotuzumab @GA-P1-lipo	Adsorption efficiency (%)	68.01±1.28 (CV=1.63%)	57.66±6.37 (CV=9.42)	67.33±1.69 (CV=2.17)	64.33±6.03	8.11
	Size (nm)	104.1±2.06 (CV=1.98)	104.9±1.10 (CV=1.37)	103.3±1.68 (CV=3.66)	104.2±1.61	1.55
	PDI	0.097±0.074	0.044±0.001	0.053±0.064	0.064±0.054	
	Zeta potential (mV)	-4.57±0.183	-5.27±0.576	-5.47±1.19	-5.10±0.78	
Rituximab @GA-P1-lipo	Adsorption efficiency (%)	67.46±1.50 (CV=1.94)	62.18±5.89 (CV=8.16)	64.05±4.16 (CV=5.62)	64.56±4.35	5.84
	Size (nm)	106.4±2.16 (CV=2.04)	104.9±5.63 (CV=5.37)	104.6±5.05 (CV=4.83)	105.3±4.02	3.82
	PDI	0.085±0.066	0.165±0.081	0.075±0.090	0.146±0.65	
	Zeta potential (mV)	-6.07±0.38	-8.50±0.49	-6.96±1.24	-7.18±1.27	

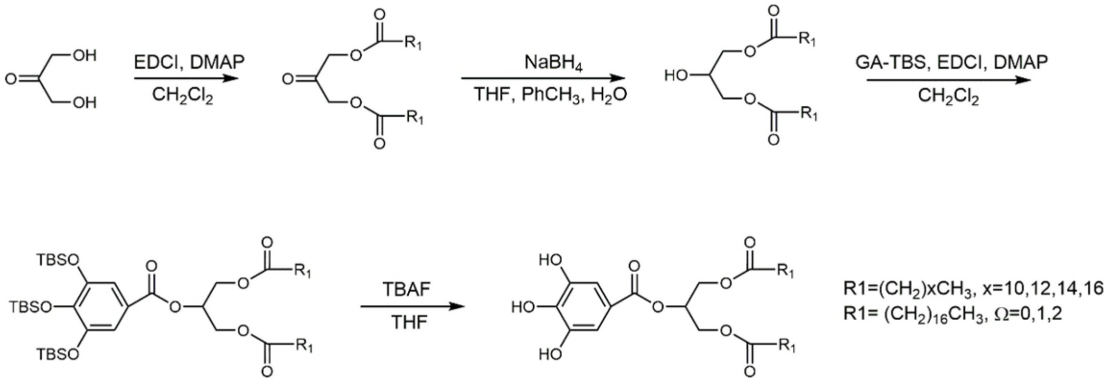

Supplementary Fig.1 Synthesis route of GA-lipid.

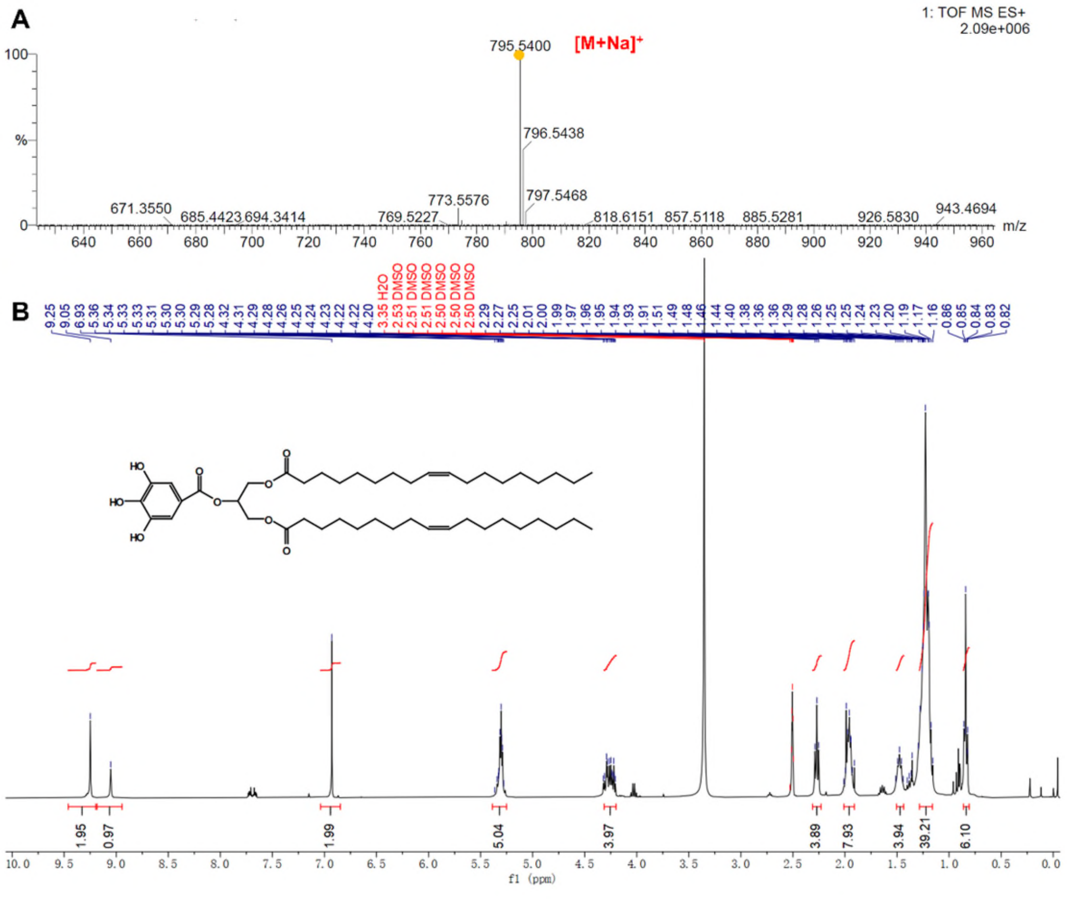

Supplementary Fig.2 Structure confirmation of GA-C18(1)-lipid. (A) MS (ESI) m/z for C46H76O9Na [M + Na]$^+$: 795.5387. (B) ^{1}H NMR (400 MHz, DMSO-d_6) δ 9.25 (s, 2H), 9.05 (s, 1H), 6.93 (s, 2H), 5.39 – 5.25 (m, 5H), 4.31 – 4.20 (m, 4H), 2.27 (t, *J* = 7.3 Hz, 4H), 2.01 – 1.91 (m, 8H), 1.47 (q, *J* = 7.1 Hz, 4H), 1.28 – 1.16 (m, 39H), 0.86 – 0.81 (m, 6H).

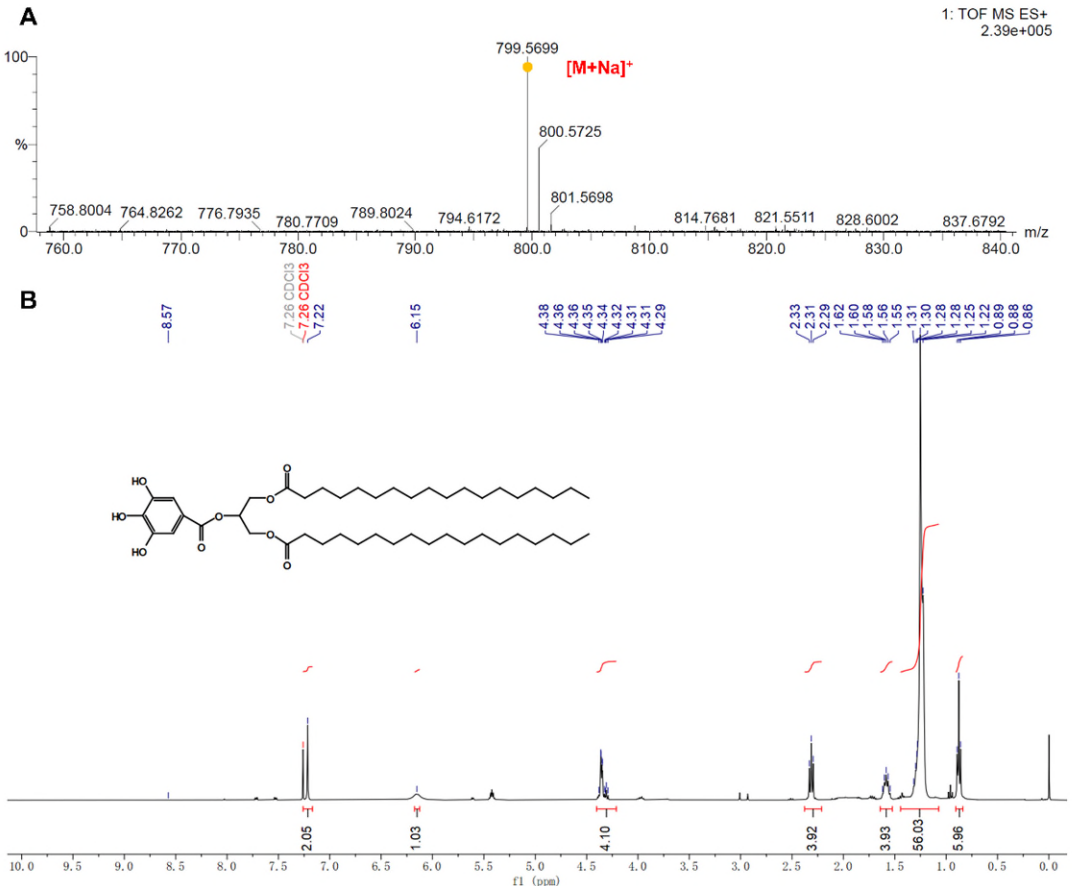

Supplementary Fig.3 Structure confirmation of GA-C18(0)-lipid. (A) MS (ESI) m/z for C46H80O9Na [M + Na]$^{+}$: 799.5699. (B) ^{1}H NMR (400 MHz, $CDCl_3$) δ 7.22 (s, 2H), 6.15 (s, 1H), 4.40 – 4.21 (m, 4H), 2.31 (t, J = 7.6 Hz, 4H), 1.58 (p, J = 7.3 Hz, 4H), 1.24 (d, J = 10.9 Hz, 56H), 0.88 (t, J = 6.8 Hz, 6H).

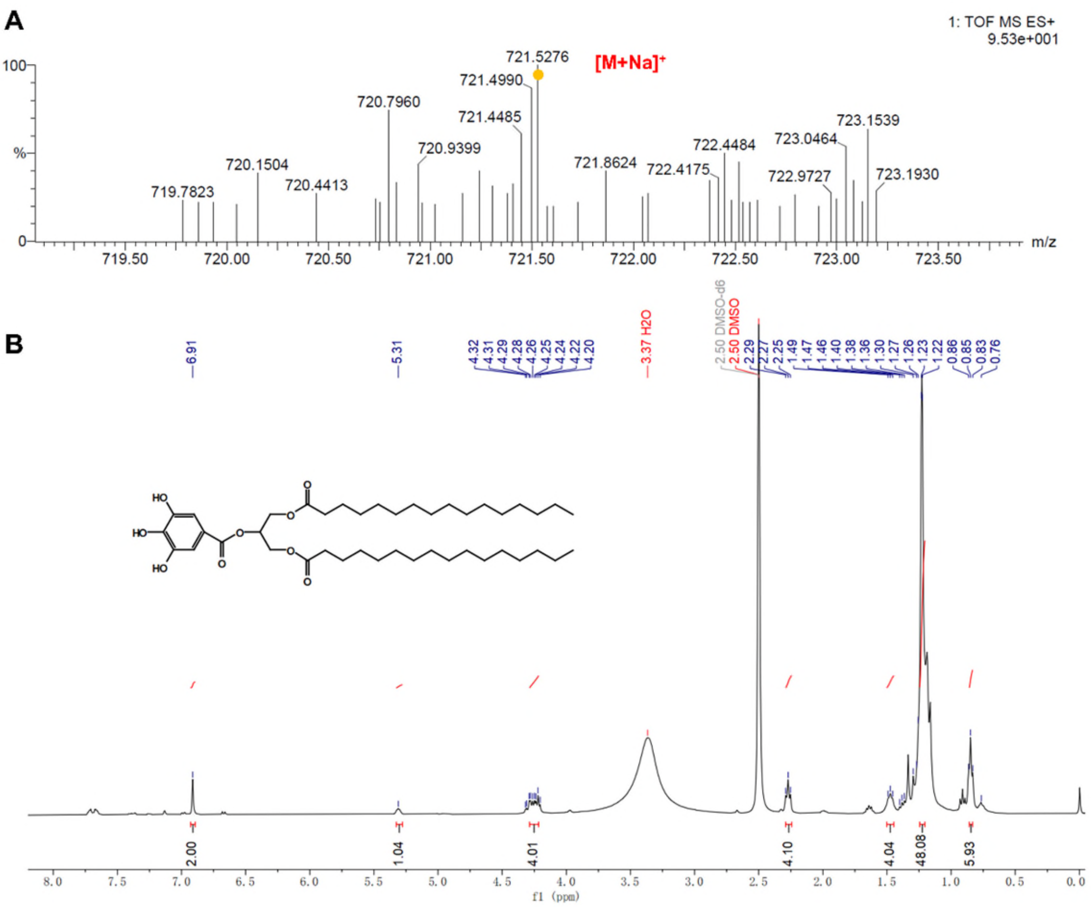

Supplementary Fig.4 Structure confirmation of GA-C16(0)-lipid. (A) MS (ESI) m/z for C42H72O9Na [M + Na]$^{+}$: 721.5255. (B) ^{1}H NMR (400 MHz, DMSO-d_6) δ 6.91 (s, 2H), 5.31 (s, 1H), 4.29 – 4.22 (m, 4H), 2.26 (d, J = 7.3 Hz, 4H), 1.50 – 1.45 (m, 4H), 1.24 – 1.20 (m, 48H), 0.85 (s, 6H).

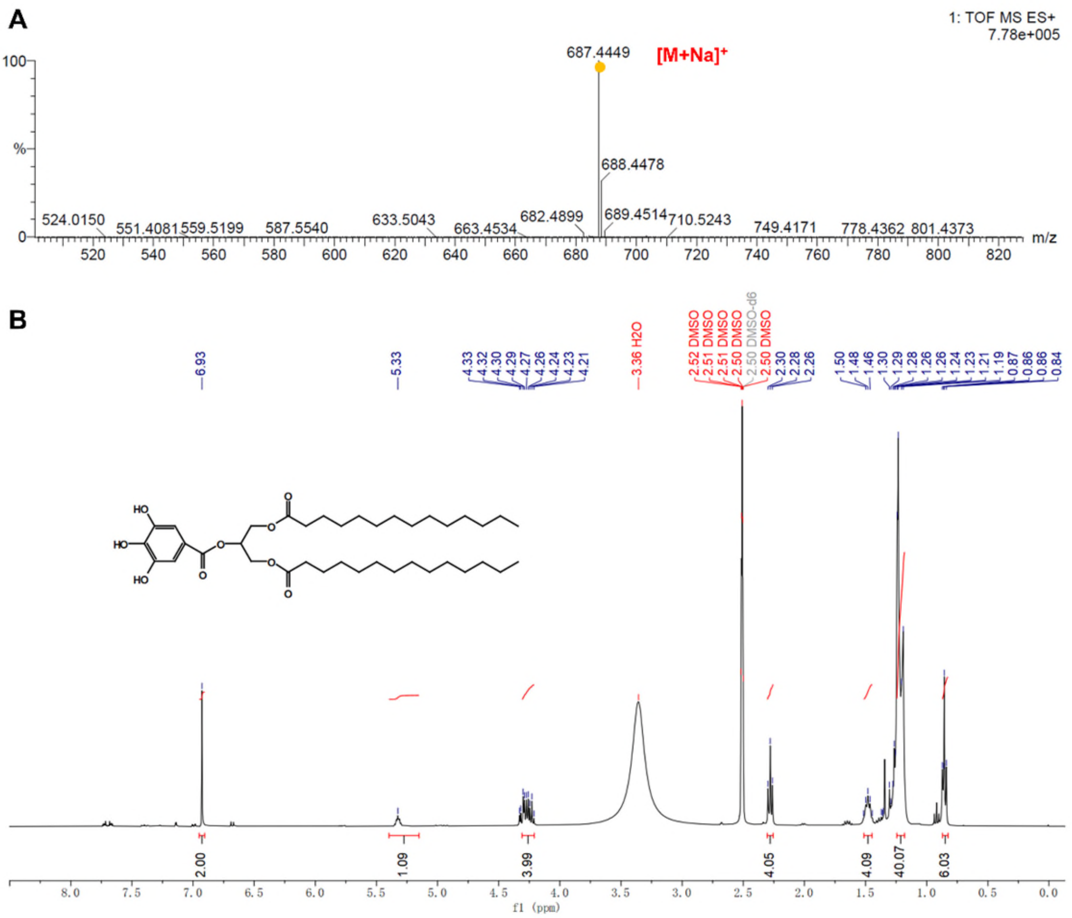

Supplementary Fig.5 Structure confirmation of GA-C14(0)-lipid. (A) MS (ESI) m/z for C38H64O9Na [M + Na]$^{+}$: 687.4448. (B) ^{1}H NMR (400 MHz, DMSO-d_6) δ 6.93 (s, 2H), 5.33 (s, 1H), 4.31 – 4.21 (m, 4H), 2.28 (t, *J* = 7.3 Hz, 4H), 1.48 (t, *J* = 7.1 Hz, 4H), 1.22 (dd, *J* = 15.1, 4.7 Hz, 40H), 0.85 (d, *J* = 6.9 Hz, 6H).

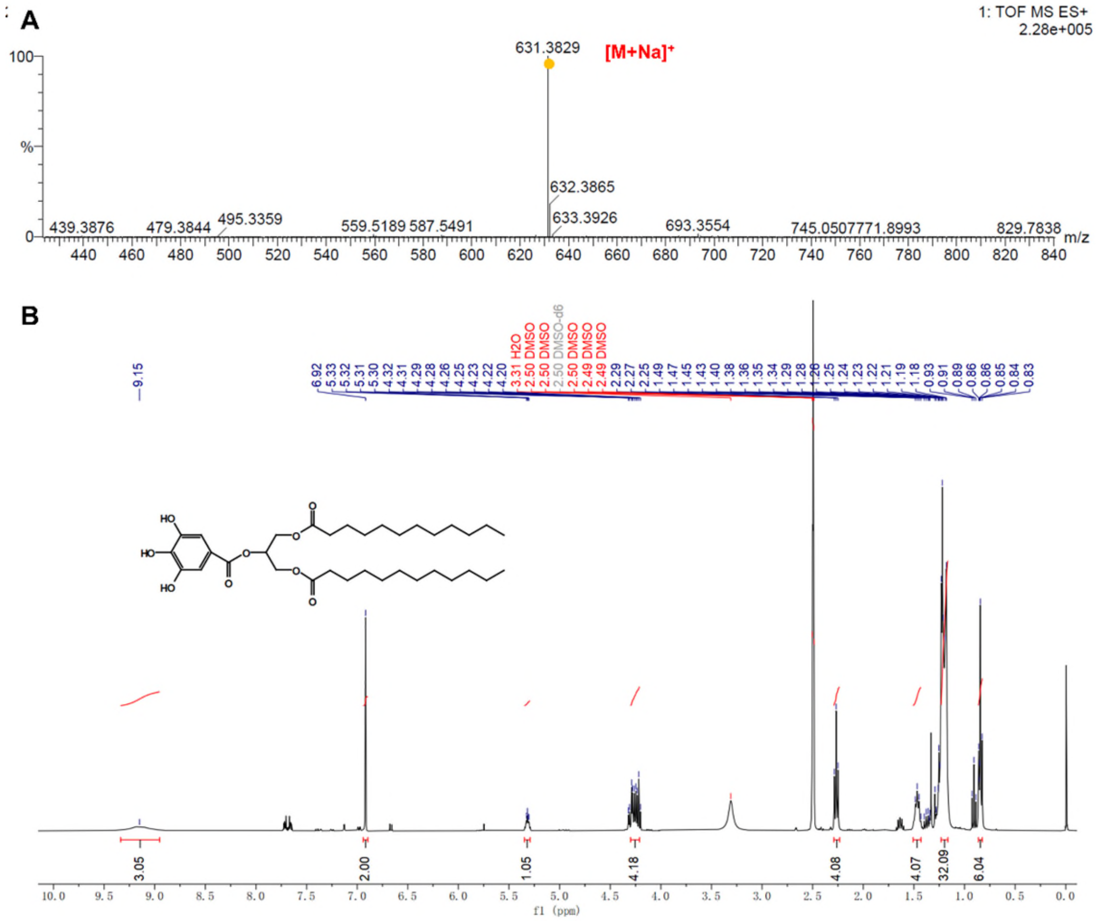

Supplementary Fig.6 Structure confirmation of GA-C12(0)-lipid. (A) MS (ESI) m/z for C34H56O9Na [M + Na]$^+$: 631.3822. (B) ^{1}H NMR (400 MHz, DMSO-d_6) δ 9.15 (s, 3H), 6.92 (s, 2H), 5.32 (dd, *J* = 6.3, 4.1 Hz, 1H), 4.30 – 4.21 (m, 4H), 2.27 (t, *J* = 7.3 Hz, 4H), 1.46 (q, *J* = 7.2 Hz, 4H), 1.23 – 1.16 (m, 32H), 0.86 – 0.82 (m, 6H).

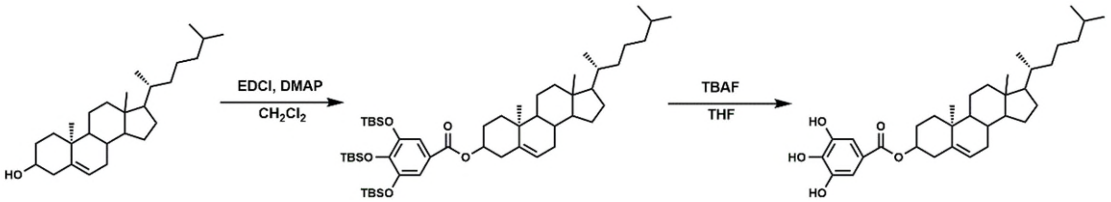

Supplementary Fig.7 Synthesis route of GA-Chol.

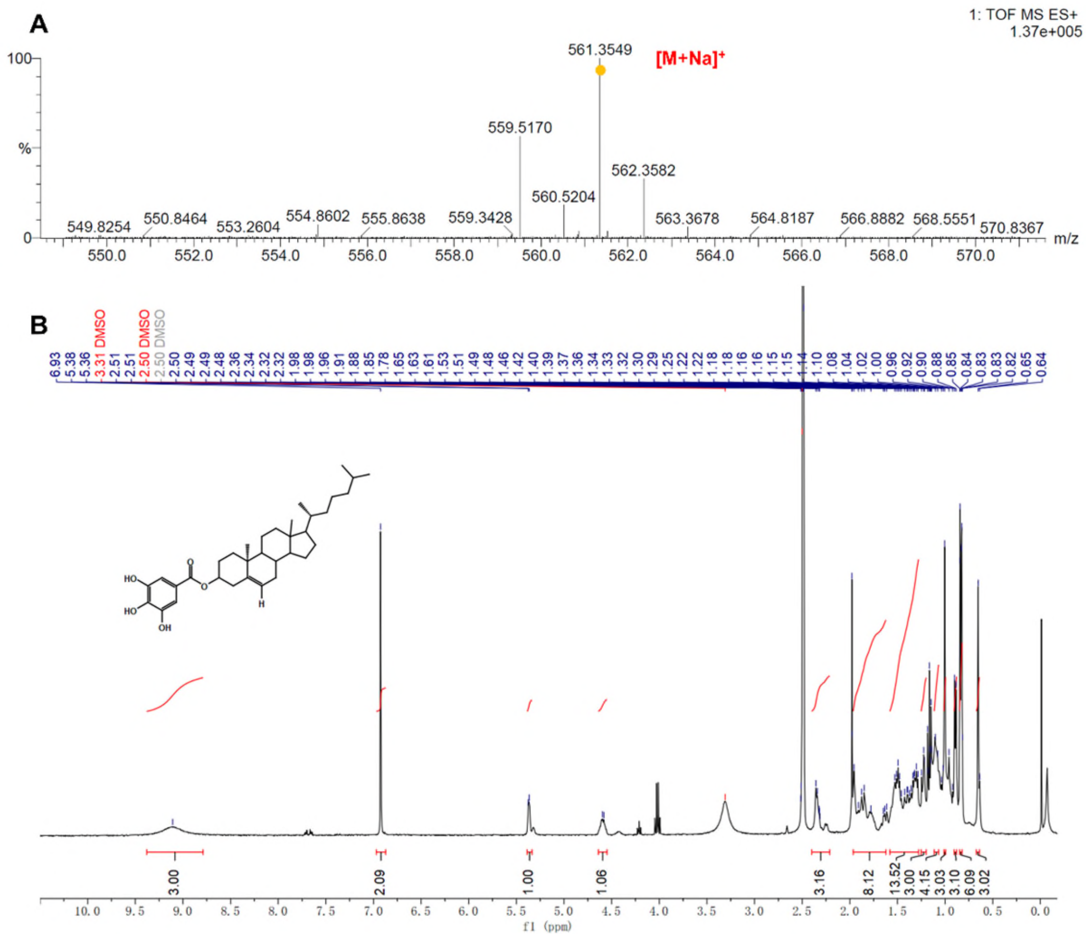

Supplementary Fig. 8 Structure confirmation of GA-Chol. (A) MS (ESI) m/z for C34H50O5Na $[M + Na]^+$: 561.3549. (B) ^{1}H NMR (400 MHz, DMSO-d_6) δ 9.12 (s, 3H), 6.94 (s, 2H), 5.38 (d, J = 4.4 Hz, 1H), 4.60 (d, J = 6.1 Hz, 1H), 2.41 – 2.22 (m, 3H), 1.98 – 1.63 (m, 8H), 1.55 – 1.30 (m, 12H), 1.25 (d, J = 8.9 Hz, 2H), 1.11 (s, 4H), 1.02 (s, 3H), 0.90 (d, J = 6.5 Hz, 3H), 0.85 (dd, J = 6.6, 2.0 Hz, 6H), 0.66 (s, 3H).

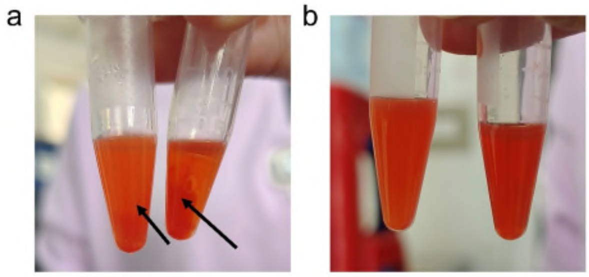

Supplementary Fig.9 The photos of GA-lipo comprising a, GA-lipid 18(1) and b, GA-P0-Chol at 4 ℃ for 7 days. Sedimentation observed in GA-lipo comprising GA-lipid18(1).

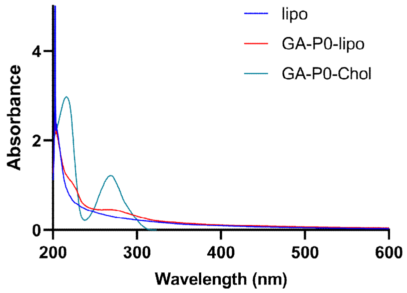

Supplementary Fig. 10 The UV spectrum of GA-P0-lipo.

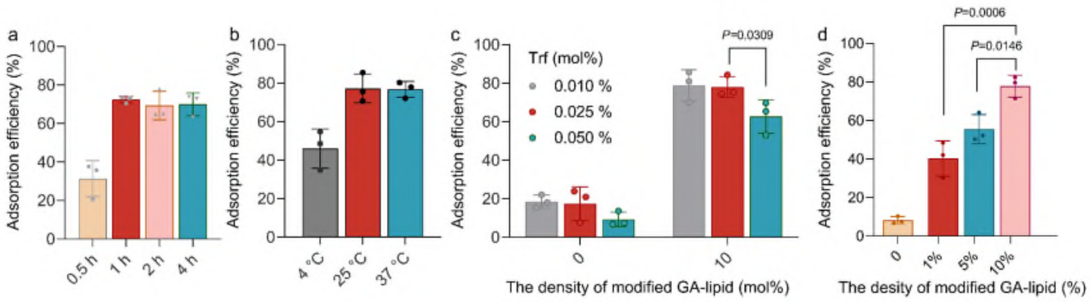

Supplementary Fig.11 The influence of incubating conditions on Trf absorption and uptake *in vitro*. a, Incubating time and **b**, Temperature of Trf and GA-lipo influence the adsorption efficiency of Trf. The mean ± s.d. is displayed from three parallel experiments (n = 3). **c**, The influence of Trf concentration and **d**, modified GA-Chol on the absorption efficiency of protein. The mean ± s.d. is displayed from three parallel experiments (n = 3). Statistical significance was analyzed by one-way ANOVA and Tukey's multiple comparisons test.

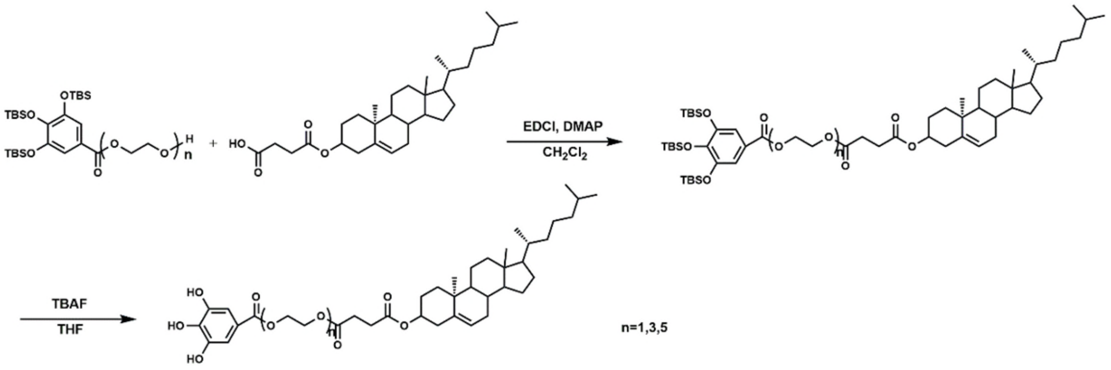

Supplementary Fig.12 Synthesis route of GA-PEG-Chol.

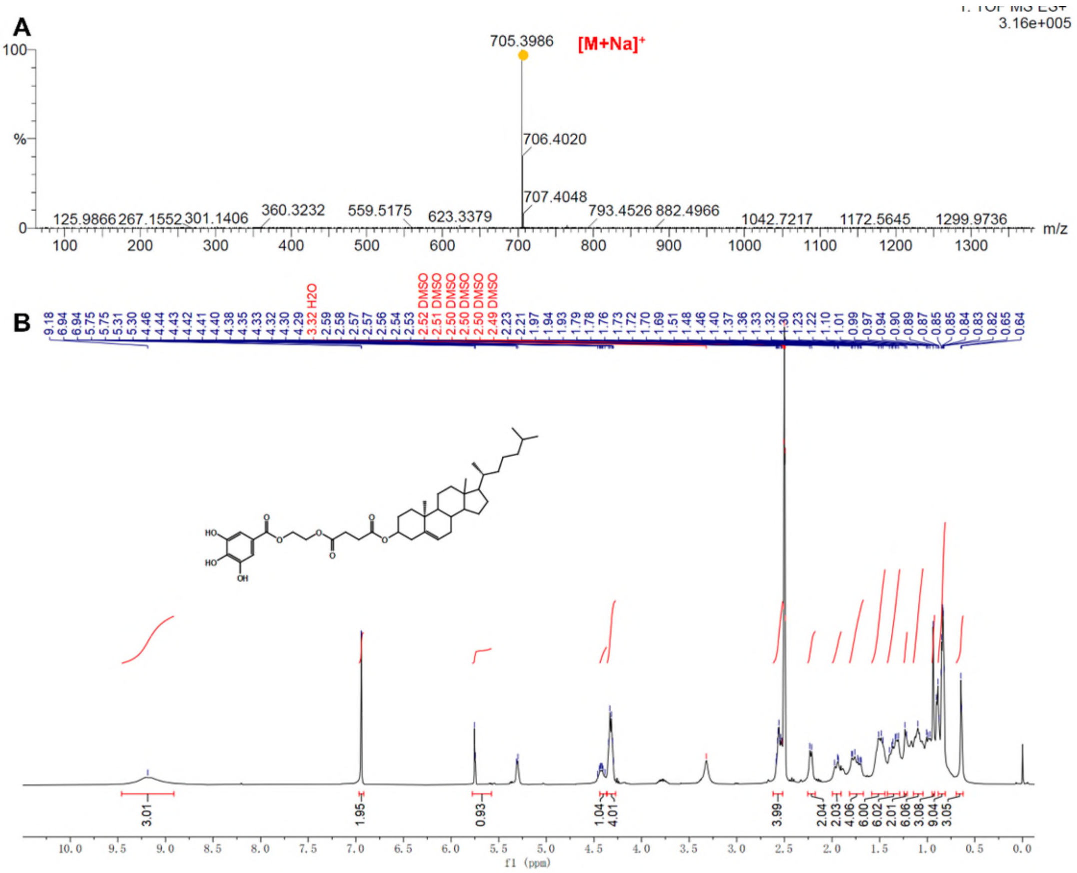

Supplementary Fig.13 Structure confirmation of GA-P1-Chol. (A) MS (ESI) m/z for C40H58O9Na [M + Na]$^+$: 705.3979. (B) ^{1}H NMR (400 MHz, DMSO-d$_6$) δ 9.18 (s, 3H), 6.94 (d, *J* = 1.4 Hz, 2H), 5.75 (d, *J* = 3.4 Hz, 1H), 4.41 (td, *J* = 8.2, 4.2 Hz, 1H), 4.32 (q, *J* = 5.4 Hz, 4H), 2.56 (h, *J* = 4.0 Hz, 4H), 2.22 (d, *J* = 8.0 Hz, 2H), 2.00 – 1.90 (m, 2H), 1.82 – 1.67 (m, 4H), 1.49 (d, *J* = 18.4 Hz, 6H), 1.35 (dt, *J* = 22.8, 8.8 Hz, 6H), 1.23 (d, *J* = 6.8 Hz, 2H), 1.10 (s, 6H), 0.94 (s, 3H), 0.84 (dt, *J* = 6.1, 2.9 Hz, 9H), 0.64 (d, *J* = 3.6 Hz, 3H).

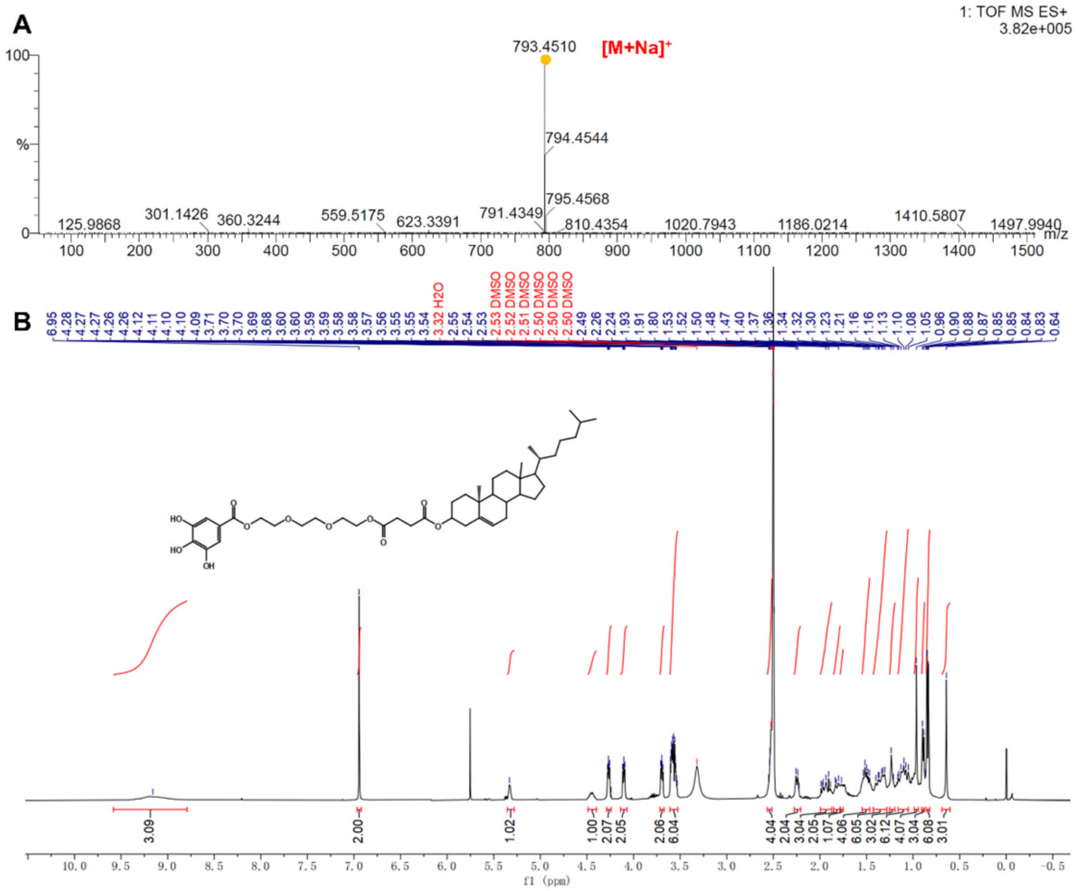

Supplementary Fig.14 Structure confirmation of GA-P3-Chol. (A) MS (ESI) m/z for C44H66O11Na [M + Na]$^+$: 793.6510. (B) ^{1}H NMR (400 MHz, DMSO-d_6) δ 9.16 (s, 3H), 6.95 (s, 2H), 5.33 (s, 1H), 4.49 – 4.40 (m, 1H), 4.29 – 4.24 (m, 2H), 4.14 – 4.07 (m, 2H), 3.72 – 3.67 (m, 2H), 3.61 – 3.53 (m, 6H), 2.52 (d, *J* = 2.9 Hz, 4H), 2.25 (d, *J* = 8.0 Hz, 2H), 2.00 – 1.87 (m, 3H), 1.85 – 1.78 (m, 2H), 1.77 (s, 1H), 1.50 (dt, *J* = 12.9, 6.8 Hz, 4H), 1.43 – 1.28 (m, 6H), 1.23 (s, 3H), 1.16 – 1.05 (m, 6H), 0.96 (s, 4H), 0.89 (d, *J* = 6.4 Hz, 3H), 0.84 (dd, *J* = 6.6, 2.0 Hz, 6H), 0.64 (s, 3H).

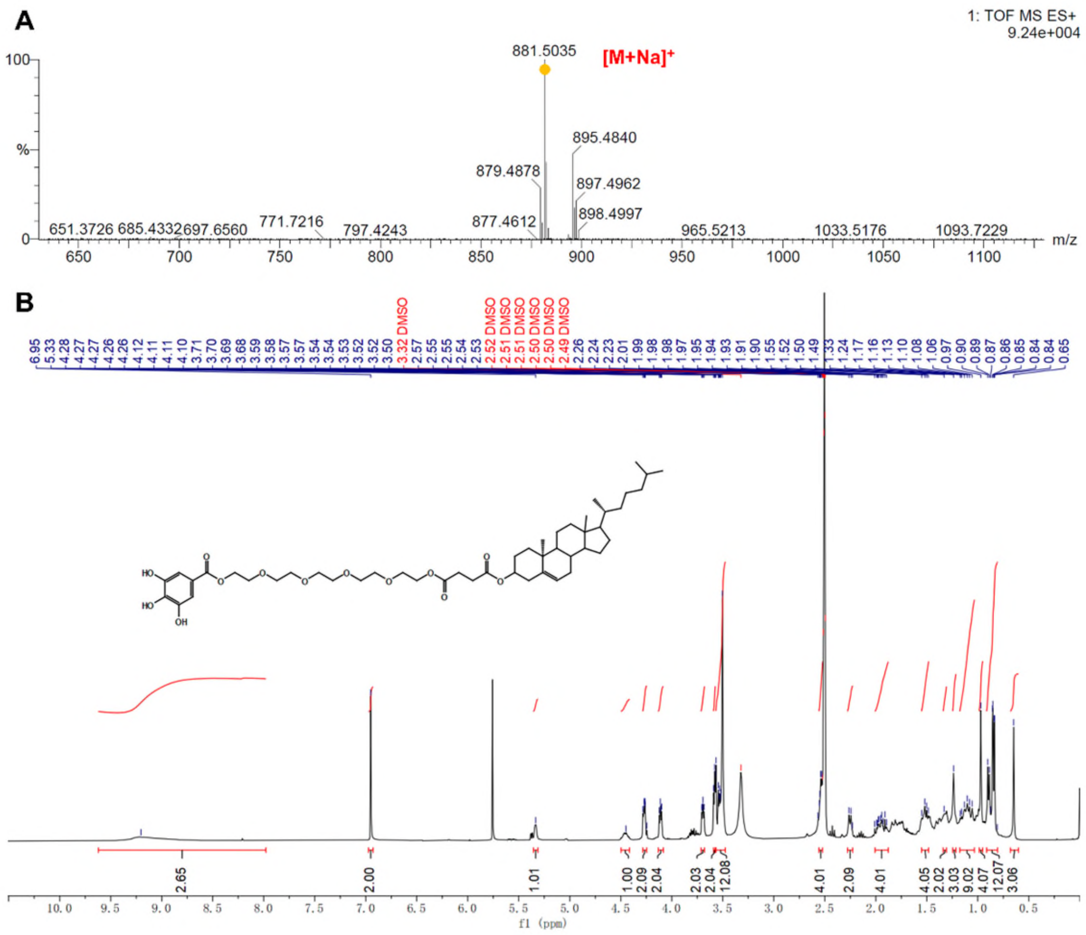

Supplementary Fig.15 Structure confirmation of GA-P5-Chol. (A) MS (ESI) m/z for C48H74O13Na [M + Na]$^+$: 881.5027. (B) ^{1}H NMR (400 MHz, DMSO-d_6) δ 9.20 (s, 3H), 6.95 (s, 2H), 5.33 (s, 1H), 4.45 (s, 1H), 4.27 (dd, *J* = 6.7, 2.6 Hz, 2H), 4.11 (dd, *J* = 5.8, 3.7 Hz, 2H), 3.70 (dd, *J* = 5.7, 3.7 Hz, 2H), 3.58 (d, *J* = 4.3 Hz, 2H), 3.57 – 3.47 (m, 12H), 2.54 (dd, *J* = 6.3, 3.1 Hz, 4H), 2.25 (d, *J* = 8.0 Hz, 2H), 2.01 – 1.88 (m, 4H), 1.55 – 1.48 (m, 4H), 1.33 (s, 2H), 1.24 (s, 3H), 1.12 (td, *J* = 17.3, 7.4 Hz, 9H), 0.97 (s, 4H), 0.91 – 0.81 (m, 12H), 0.65 (s, 3H).

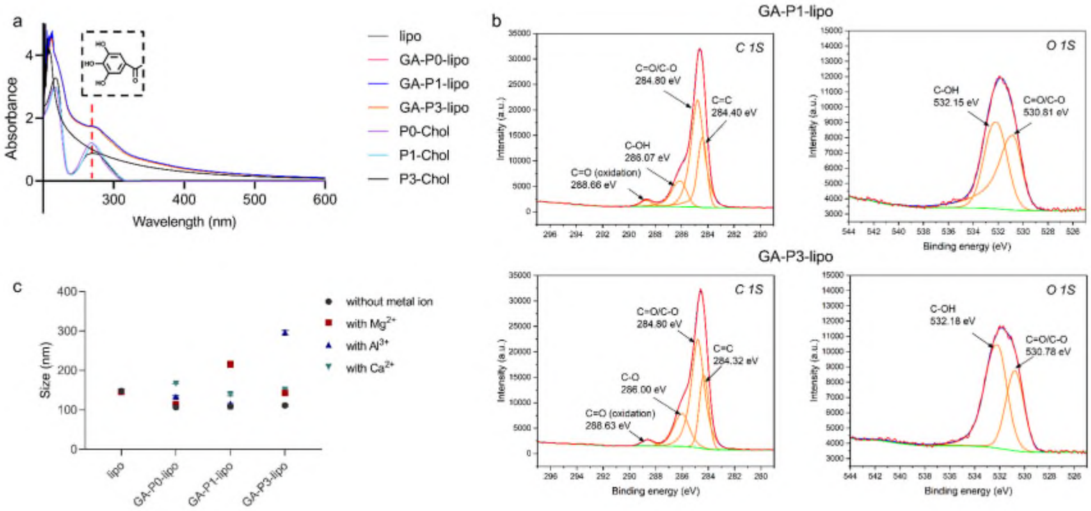

Supplementary Fig.16 The polyphenols in the bilayer of liposomes can exposure on the surface. **a**, UV absorption spectra of lipo and GA-P-lipos. The result indicated that the galloyl acid modified lipids were successfully incorporated into the phospholipid bilayer. **b**, High-resolution individual XPS spectra (*C 1s* and *O 1s*) of GA-P1-lipo and GA-P0-lipo. **c**, The exposure polyphenols on the surface of GA-P-lipo can complex with metal ion, making the size of GA-lipo increase. The sizes of lipo and GA-fatty acid lipid after incubating with Mg^{2+}, Al^{3+} and Ca^{2+}. The mean ± s.d. is displayed from three parallel experiments (n = 3). The results showed the successful exposure of galloyl moieties on the surface of all GA-P-lipo variants.

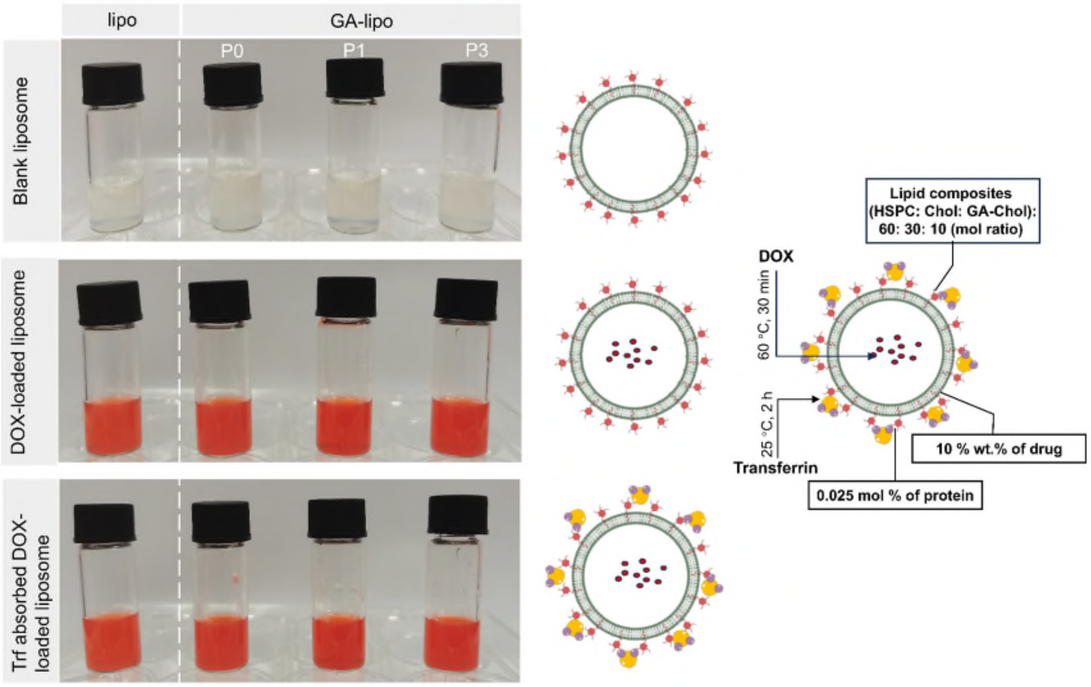

Supplementary Fig.17 The photos of different formulations.

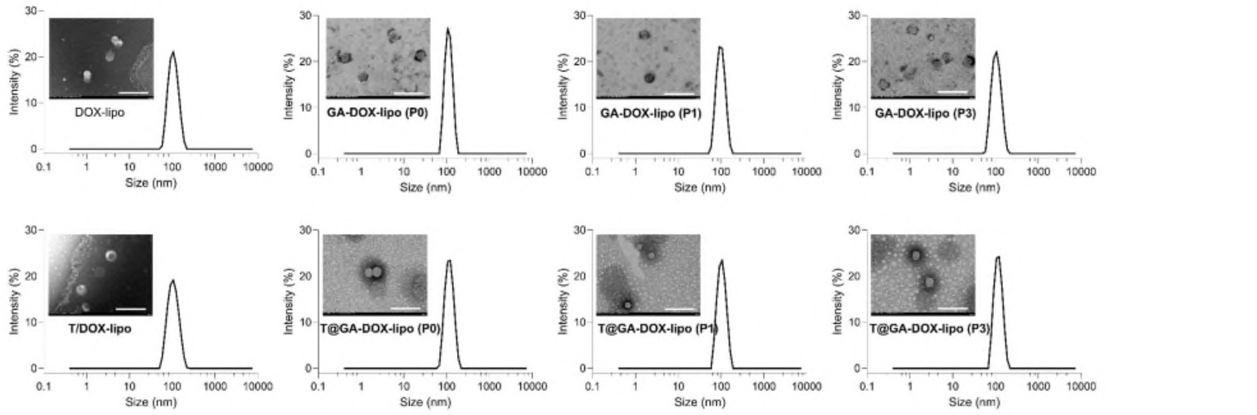

Supplementary Fig.18 Electron microscopy of DOX-loaded liposomes and Trf adsorbed DOX-loaded liposomes. TEM image and size distribution of liposomes. Scale bar: 500 nm. Experiments were repeated three times.

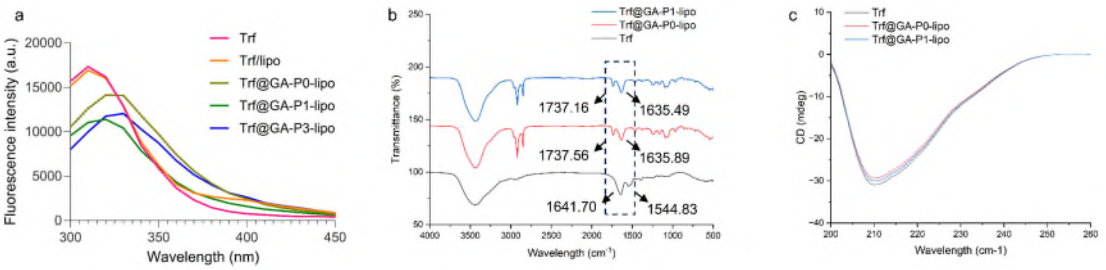

Supplementary Fig.19 Secondary structure of transferrin adsorbed on GA-lipo. a, Trf Trp quenching after adsorption to GA-lipo. b, FT-IR spectra and c, CD spectra of Trf and Trf@GA-lipo.

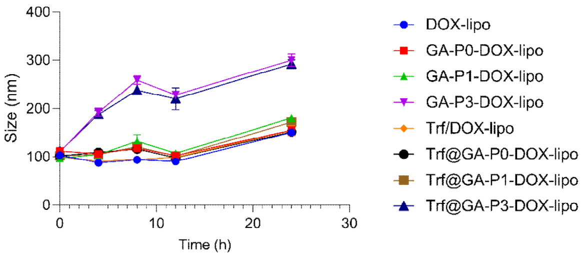

Supplementary Fig.20 The size change of DOX-loaded liposomes in PBS with 10% FBS at 37 °C. Notable increase in particle size of Trf@GA-P0-DOX-lipo and GA-P0-DOX-lipo during storage at 37 °C. The mean ± s.d. is displayed from three parallel experiments (n = 3).

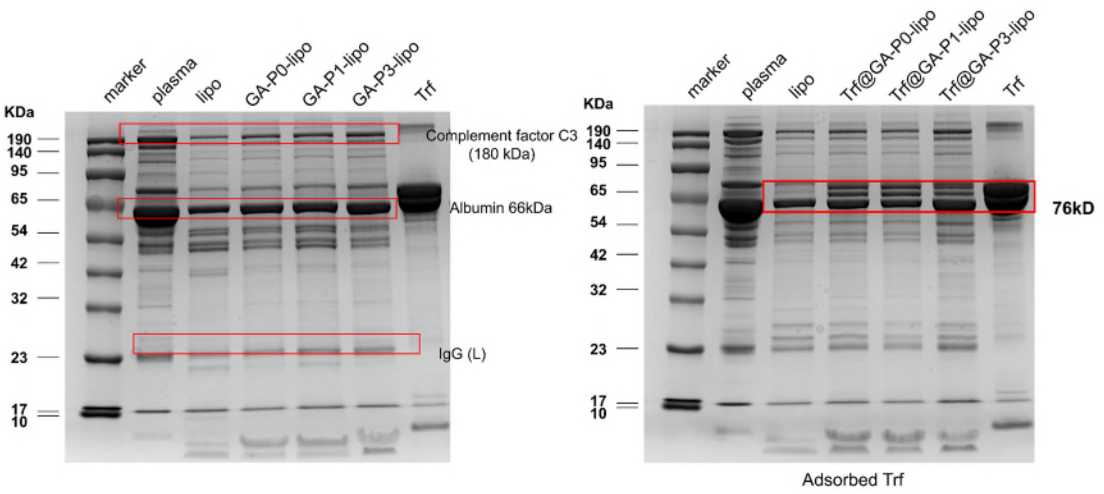

Supplementary Fig.21 Adsorbed Trf remained on the surface of GA-lipo during the formation of protein corona. Liposomes were incubated with 50% mice plasma at 37 ℃ for 2 h, isolated via centrifugation and washed three times to remove unbound proteins. Hard corona proteins were desorbed from the particles using 2% SDS, separated by SDS-PAGE and stained with Evans Blue. Experiments were repeated three times.

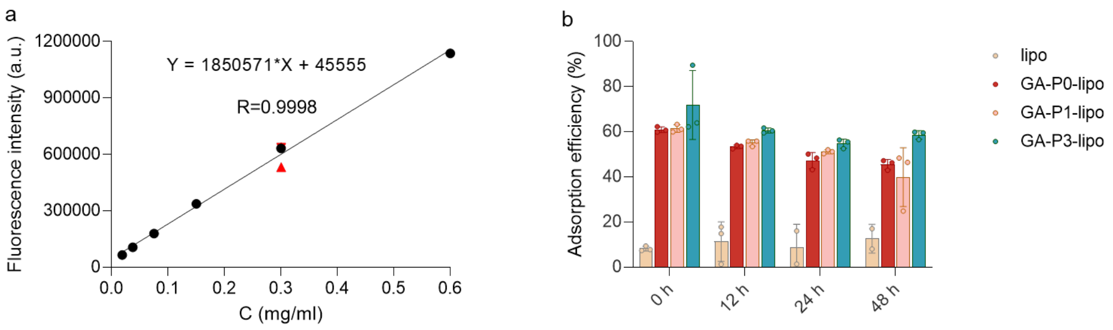

Supplementary Fig.22 Adsorbed Trf on GA-lipo stable in the plasma. a, The standard curve of Trf-FITC. **b**, Liposomes were incubated with 50% mice plasma at 37 ℃ for 2 h, centrifugation and measured the fluorescence intensity of FITC in the supernatant. The mean ± s.d. is displayed from three parallel experiments (n = 3).

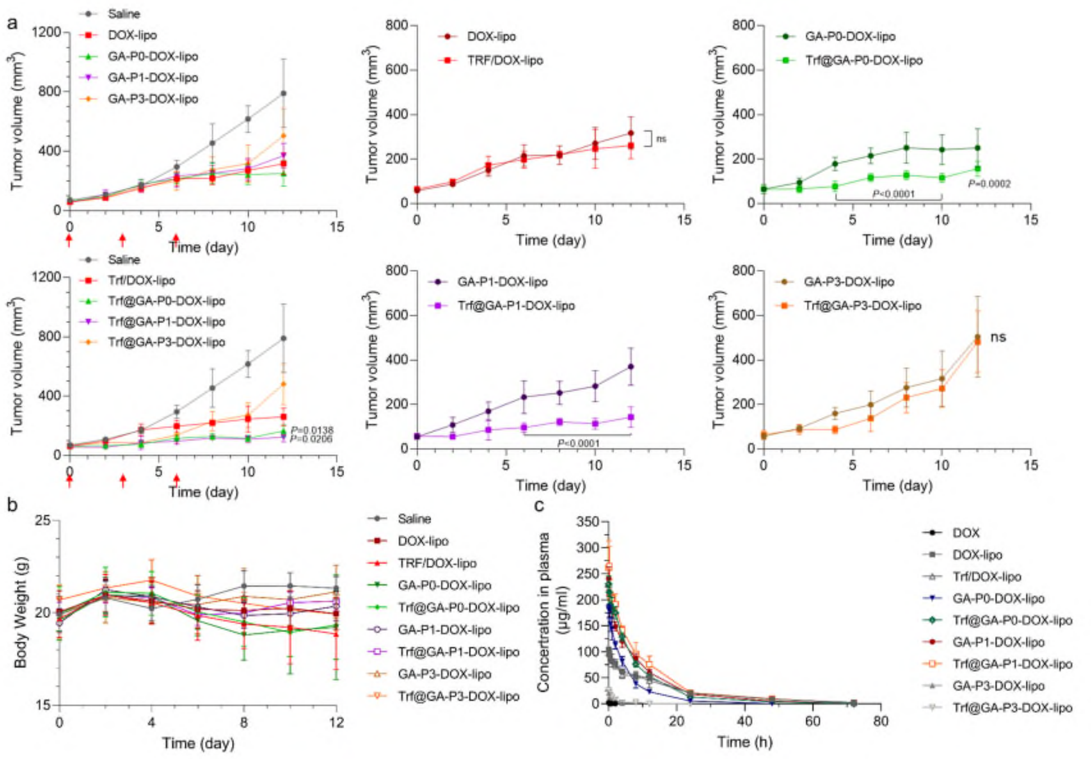

Supplementary Fig.23 The treatment efficacy of Trf@GA-lipo. **a**, The dosage of different liposomes was 3 mg/kg DOX. The tumor volume curve of different groups. The mean ± s.d. is displayed from five parallel mice (*n* = 5). Statistical significance was analyzed by two-way ANOVA and Tukey's multiple comparisons test. **b**, Body weight of different groups. The mean ± s.d. is displayed from five parallel mice (n = 5). **c**, Plasma concentration–time profiles of Trf@GA-lipo formulations after intravenous administration (*n* = 3 biologically independent samples, mean ± s.d.).

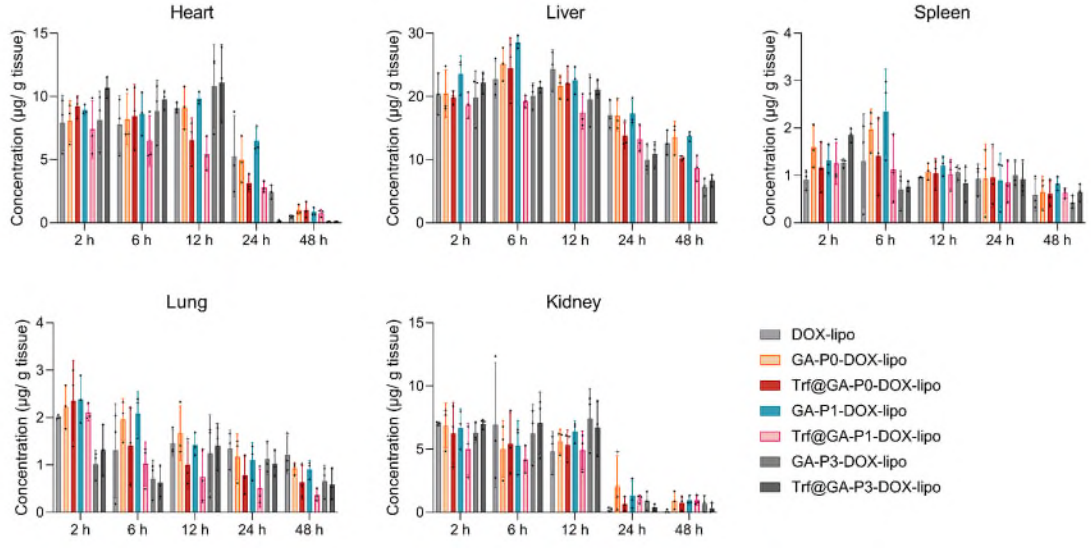

Supplementary Fig.24 In vivo biodistribution of the 4T1 tumor-bearing mice at 2, 6, 12, 24 and 48 h after i.v. administration to mice with DOX-loaded liposomes at a DOX equivalent dose of 10 mg/kg (n = 3 biologically independent samples, mean ± s.d.).

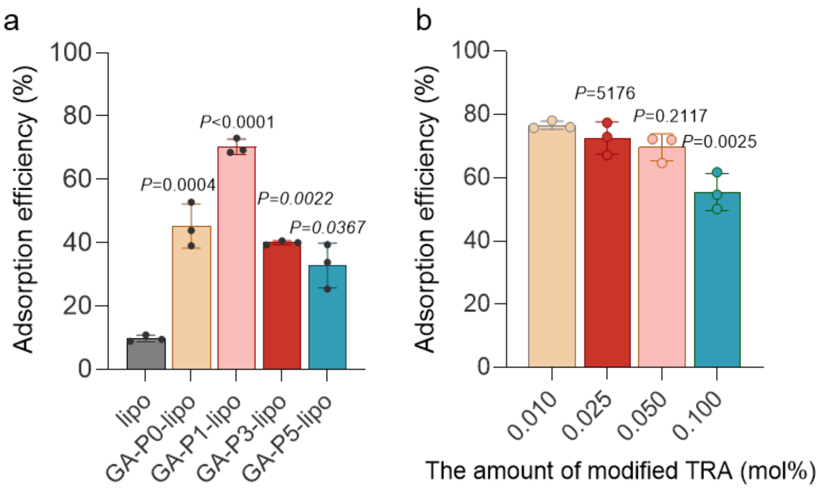

Supplementary Fig.25 The influence of incubating conditions on TRA absorption. a, The types of GA-Chol, **b**, The amount of adsorbed TRA influence the adsorption efficiency of TRA. The mean ± s.d. is displayed from three parallel experiments (n = 3). Statistical significance was analyzed by one-way ANOVA and Tukey's multiple comparisons test.

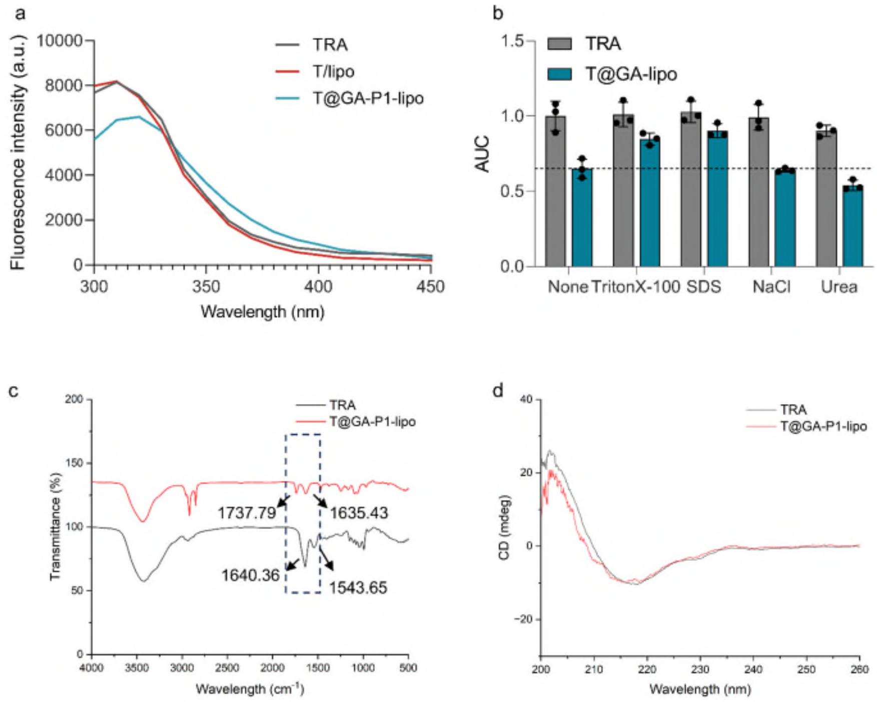

Supplementary Fig.26 The change of TRA Trp quenching after GA-lipo incubation with different chemical agents. a, TRA Trp quenching in T@GA-lipo. The excitation and emission wavelengths of λex = 280 nm and λem= 300-450 nm, respectively. TRA Trp quenching after adsorption to GA-lipo. **b**, TRA Trp quenching recovery after T@GA-lipo incubation with 100 mM TritonX-100, SDS, NaCl and Urea for 4 h. AUC normalized to folded TRA. The mean ± s.d. is displayed from three parallel experiments (n = 3).**c**, FT-IR spectra and d, CD spectra of TRA and T@GA-lipo.

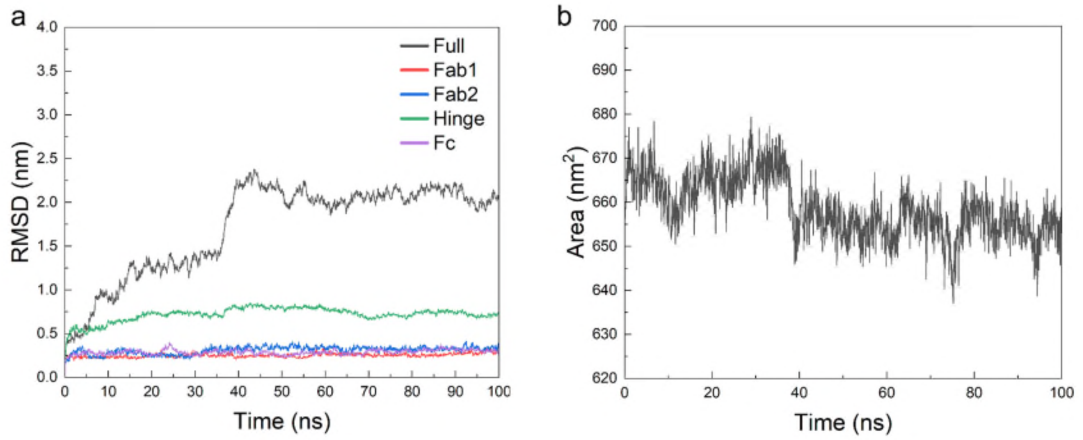

Supplementary Fig.27 Conformational stability of trastuzumab during MD simulation. a, Time-evolution of backbone RMSD (root-mean-square deviation) for the full antibody (black) and its sub-domains (Fab1, Fab2, hinge, Fc). b, Solvent-accessible surface area (SASA) of the full antibody as a function of time. RMSD and SASA profiles (Supplementary Fig. 27a-b) show <0.6 nm deviation for individual domains and stable solvent accessibility, confirming that adsorption does not distort the tertiary structure—an essential prerequisite for preserving antigen recognition.

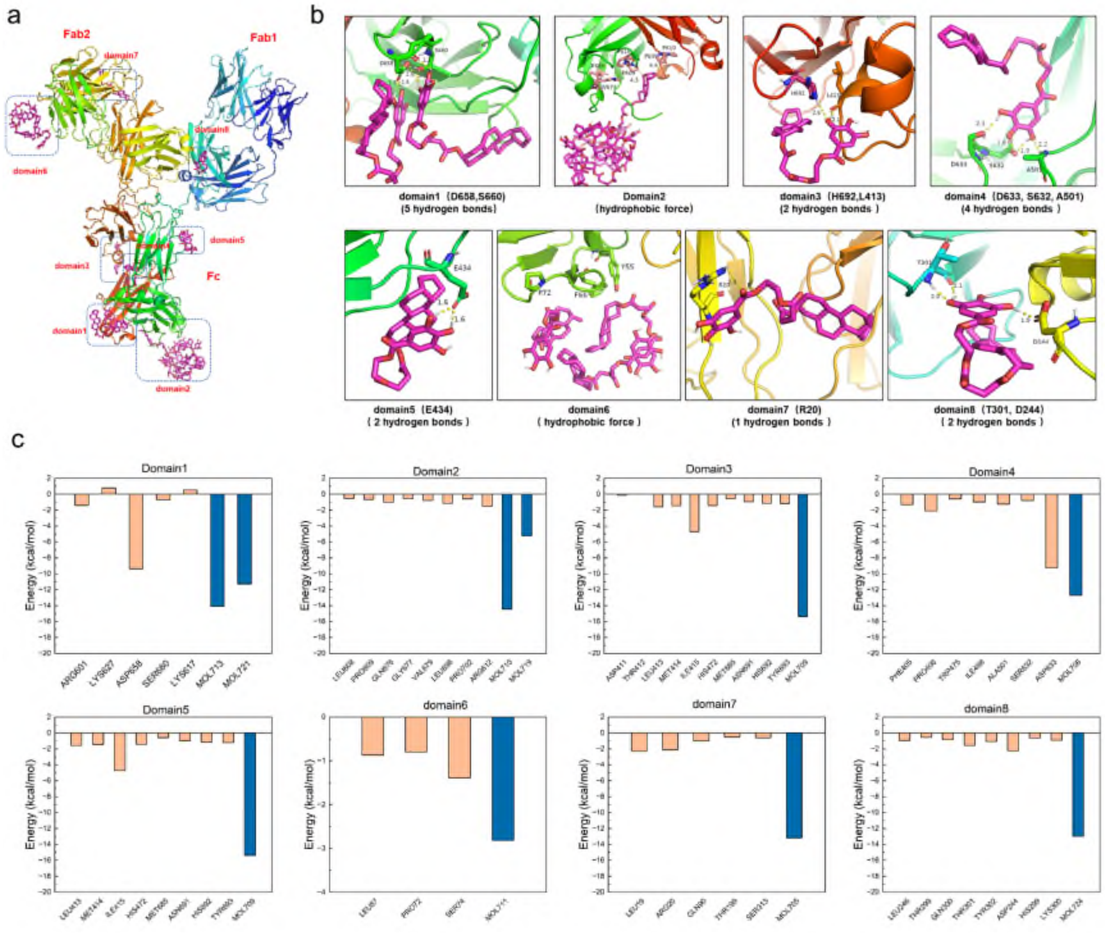

Supplementary Fig.28 Molecular interaction analysis between trastuzumab and GA-P1-Chol based on molecular dynamics simulations. a, Overall docking model of trastuzumab and GA-P1-Chol, showing the spatial location of eight identified binding domains (domains 1-8) across Fab1,

Fab2, and Fc regions. GA-P1-Chol is shown in magenta; individual antibody chains are colored distinctly. b, Representative close-up views of GA-P1-Chol interactions within each domain. Key residues involved in hydrogen bonding (yellow dashed lines) or hydrophobic interactions are labeled. Domain1 (D658, S660): 5 hydrogen bonds; Domain2: hydrophobic interactions; Domain3 (H692, L413): 2 hydrogen bonds; Domain4 (D633, S632, A501): 4 hydrogen bonds; Domain5 (E434): 2 hydrogen bonds; Domain6: hydrophobic interactions; Domain7 (R20): 1 hydrogen bond; Domain8 (T301, D244): 2 hydrogen bonds. c, Binding free energy decomposition of GA-P1-Chol to each domain based on per-residue energy contribution analysis. Bar graphs show the energy contribution (kcal/mol) of key residues; negative values indicate favorable interactions.

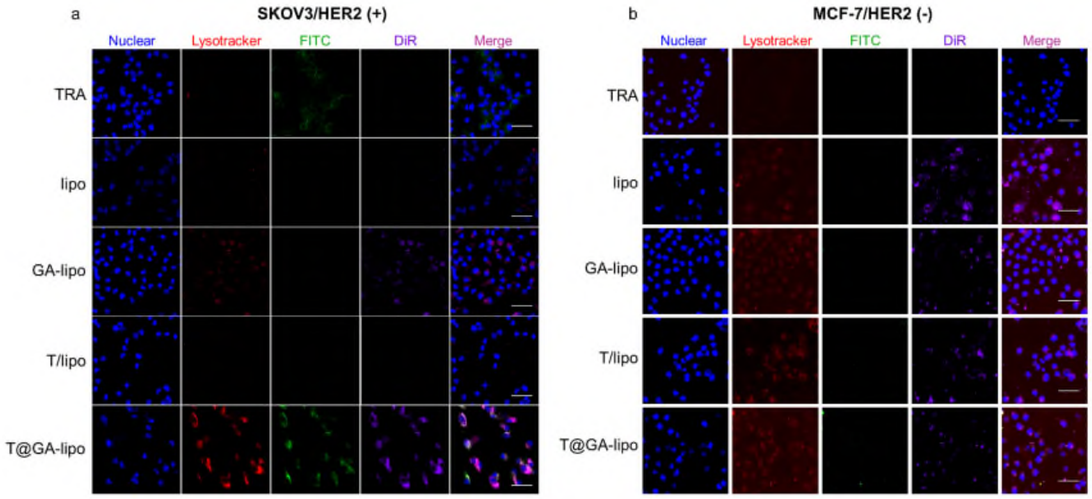

Supplementary Fig.29 Lysosome colocalization experiments. a, Adsorption of TRA to GA-lipo improves uptake on HER2 positive SKOV3 cells. Confocal analysis. After incubation of SKOV3 with different liposomes for 8 h, SKOV3 were stained with Lyso Tracker Red to lable Lysosome. Fluorescence signal of liposome is displayed with DiR. Trastuzumab is labeled with FITC. Scale bar: 50 μm. **b**, Adsorption of TRA to GA-lipo have no influence on uptake on HER2 negative MCF-7 cells. Confocal analysis. After incubation of MCF-7 with different liposomes for 8 h, MCF-7 were stained with Lyso Tracker Red to lable Lysosome. Fluorescence signal of liposome is displayed with DiR. Trastuzumab is labeled with FITC. Scale bar: 50 μm. Experiments were repeated three times.

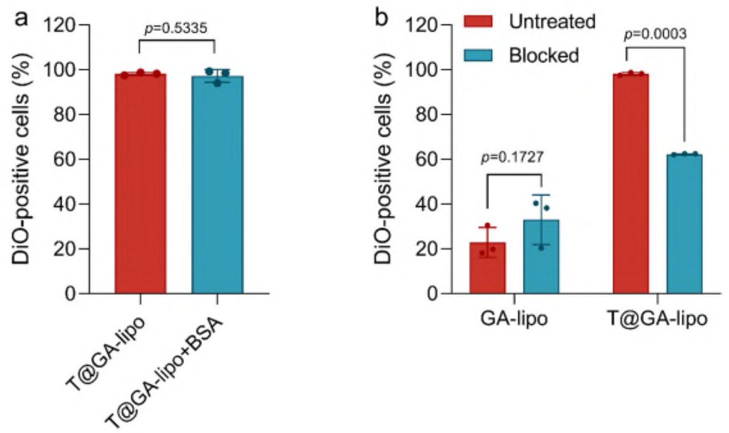

Supplementary Fig.30 Receptor-blocking assay validating HER2-specific uptake of T@GA-lipo. a, The influence of blocked T@GA-lipo with BSA on the the uptake of SKOV3 cells. T@GA-lipo was prepared by incubating T@GA-lipo with BSA at 25 ℃ for 1 h. The incubation DiO concentration was 5 μg/ml. b, SKOV3 cells were incubated with DiO-labeled GA-lipo or T@GA-lipo (5 μg/mL, 8 h). To assess receptor specificity, SKOV3 cells were pre-treated with excess anti-HER2 antibody (30 min) to block HER2 receptors prior to T@GA-lipo incubation. Cellular uptake was quantified by flow cytometry and expressed as the percentage of DiO-positive cells. Data are presented as mean ± s.d. from three independent experiments (n = 3). Statistical significance was determined by one-way ANOVA.

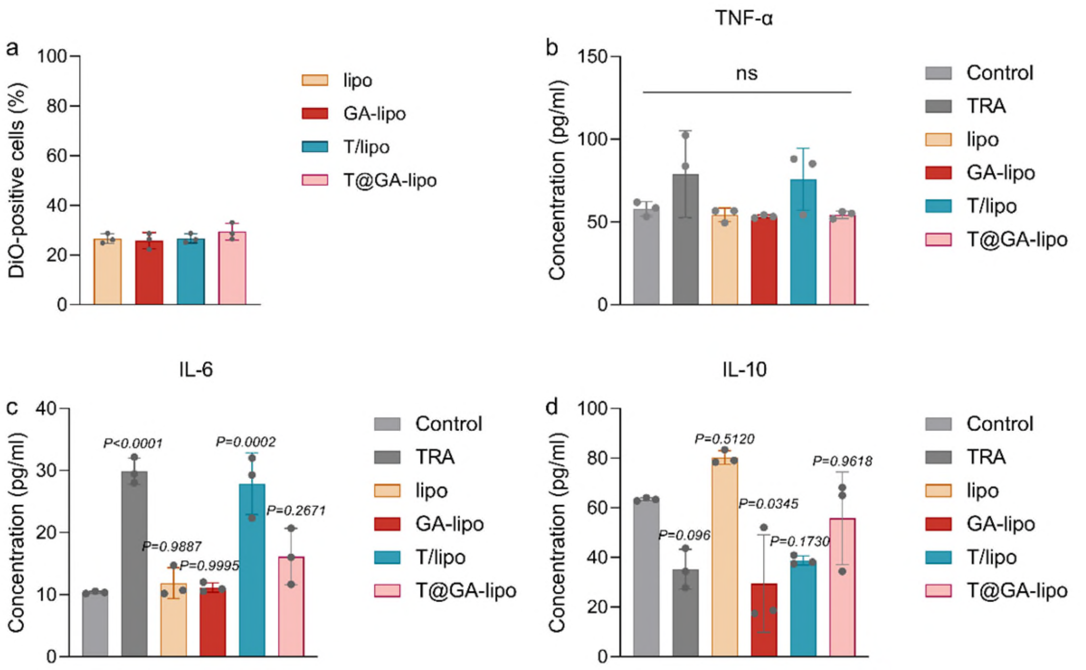

Supplementary Fig.31 Evaluation of non-specific uptake and cytokine secretion in Fc receptor-expressing macrophages. THP-1-derived macrophages were used to assess the non-specific uptake and immunostimulatory effects of different liposome formulations. a, Flow cytometry analysis of DiO-labeled liposome uptake by THP-1 macrophages. Cells were incubated with various liposomes (DiO-labeled lipo, GA-lipo, T/lipo, or T@GA-lipo; 5 μg/mL) for 12 h in complete medium. No significant increase in uptake was observed with T@GA-lipo compared to

control groups. (n = 3 biologically independent samples) b-d, Cytokine secretion profiles of THP-1 macrophages after 24 h incubation with trastuzumab (TRA), non-targeted lipo, GA-lipo, T/lipo, or T@GA-lipo. The concentrations of b, TNF-α, c, IL-6, and d, IL-10 were measured by ELISA. Data are shown as mean ± s.d. from three independent experiments (n = 3). Statistical significance was determined by one-way ANOVA; ns, not significant.

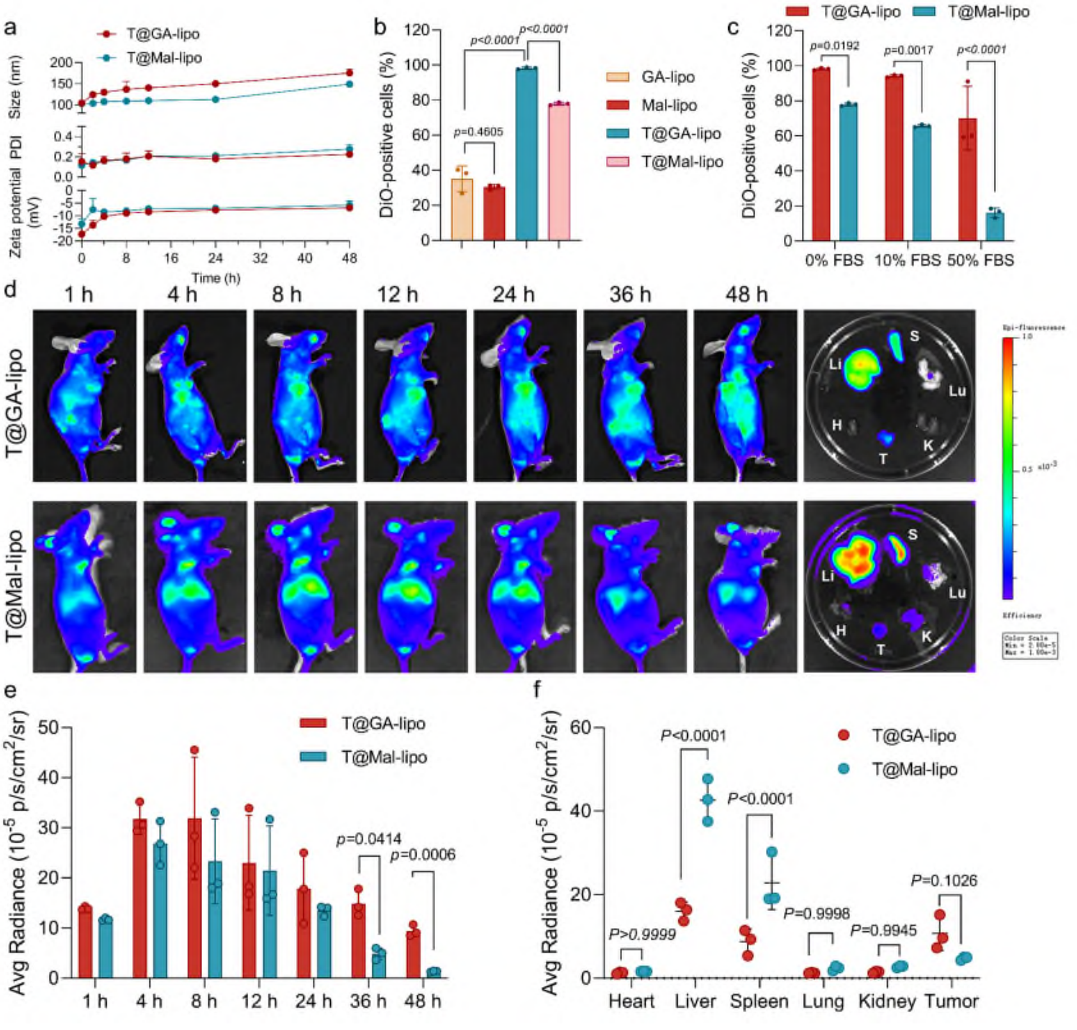

Supplementary Fig.32 Side-by-side comparison of GA-mediated adsorption and maleimide-thiol covalent conjugation. a, Time-dependent size, PDI and ζ-potential of T@GA-lipo and T@Mal-lipo in PBS + 10% FBS at 37 °C (mean ± s.d., n = 3 technical replicates). b, SKOV3 uptake of DiO-labelled liposomes after 12 h (0% FBS) (mean ± s.d., n = 3 biologically independent samples). c, Uptake at 10% and 50% FBS (12 h) determined by flow cytometry (mean ± s.d., n = 3 biologically independent samples). Statistical significance of (b) and (c) was analyzed by one-way ANOVA and Tukey's multiple comparisons test. d, In vivo fluorescence imaging of DiR labeled liposomes in tumor at different determined times after injection administration and Ex vivo fluorescence imaging of major organs obtained from the mice at 48 h post-injection. Three biological replicates were measured (n = 3). e, Semi-quantitative tumor radiance over time (mean ± s.d., n = 3). f, Fluorescence intensities of excised organs at 48 h (mean ± s.d., n = 3; one-way ANOVA with multiple-comparison post-test).

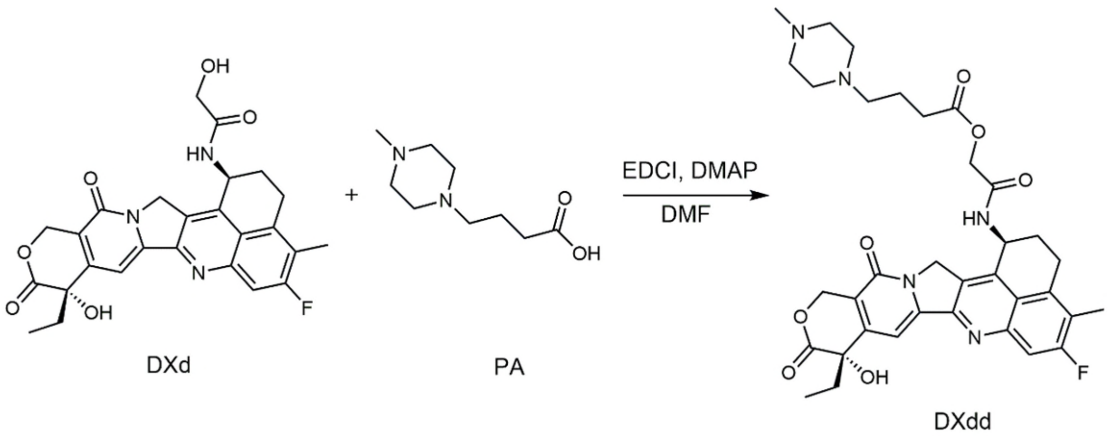

Supplementary Fig.33 Synthesis route of DXdd.

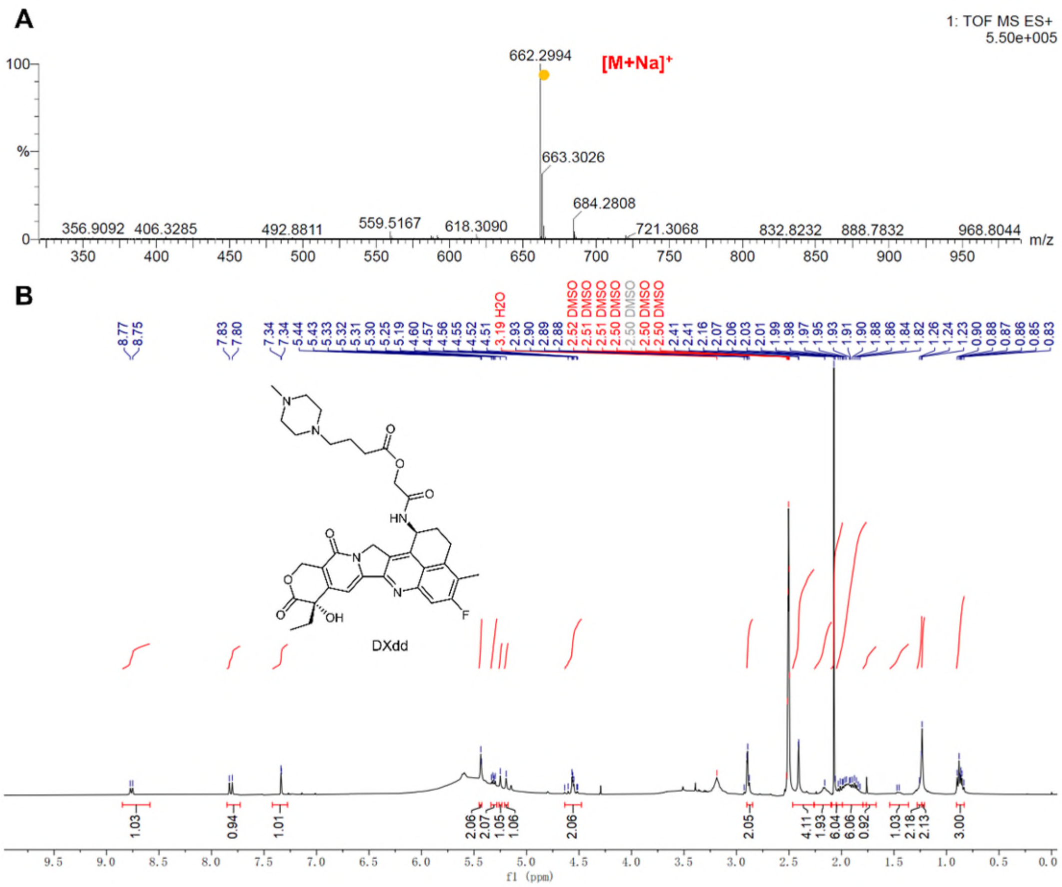

Supplementary Fig.34 Structure confirmation of DXdd. (A) MS (ESI) m/z for C35H41N5O7FNa $[M + Na]^+$: 662.2994. (B) ^{1}H NMR (400 MHz, DMSO-d_6) δ 8.76 (d, J = 8.7 Hz, 1H), 7.82 (d, J = 11.0 Hz, 1H), 7.34 (d, J = 1.7 Hz, 1H), 5.43 (d, J = 3.1 Hz, 2H), 5.32 (dd, J = 9.4, 4.7 Hz, 2H), 5.25 (s, 1H), 5.19 (s, 1H), 4.64 – 4.48 (m, 2H), 2.89 (t, J = 4.5 Hz, 2H), 2.43 (s, 4H), 2.20 (d, J = 31.6 Hz, 2H), 2.07 (s, 6H), 1.91 (ddt, J = 22.5, 14.5, 6.5 Hz, 6H), 1.76 (s, 1H), 1.46 (d, J = 8.5 Hz, 1H), 1.24 (s, 2H), 1.23 (s, 2H), 0.87 (dt, J = 11.3, 7.1 Hz, 3H).

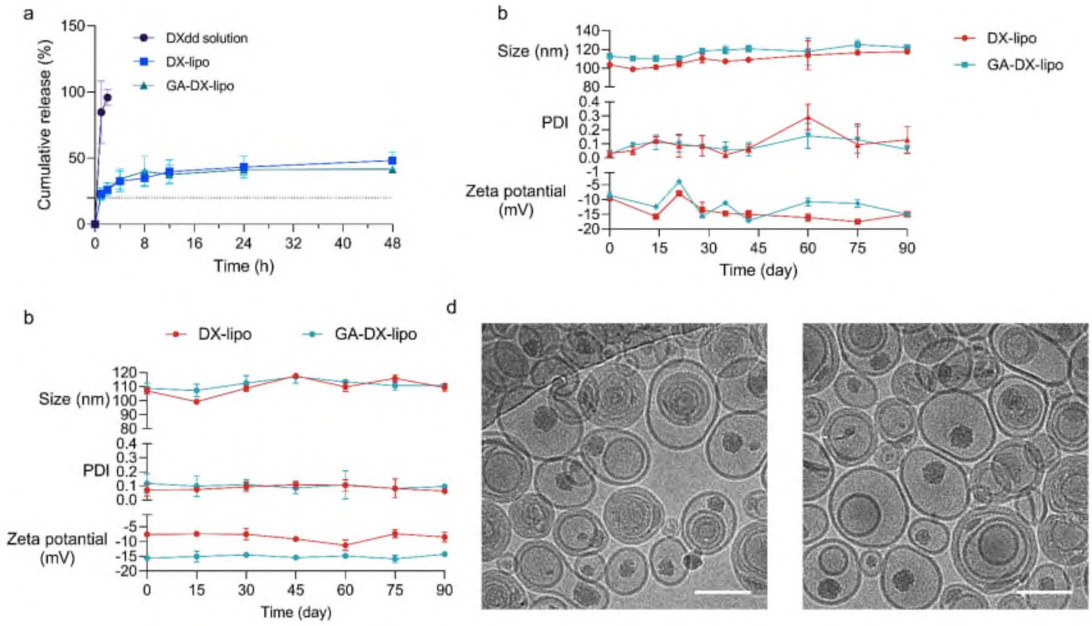

Supplementary Fig.35 Release profile and long-term storage stability of DXdd-loaded liposomes. a, Cumulative DXdd release from DXdd solution, DX-lipo, and GA-DX-lipo in PBS at 37 °C. (b, c) Size, PDI, and zeta potential of DX-lipo and GA-DX-lipo stored at (b) 4 °C and (c) 25 °C for 90 days (mean ± s.d., n = 3 technical replicates). d, Cryo-TEM images of GA-DX-lipo: left, freshly prepared; right, after 3 months at 25 °C. Scale bars: 100 nm.

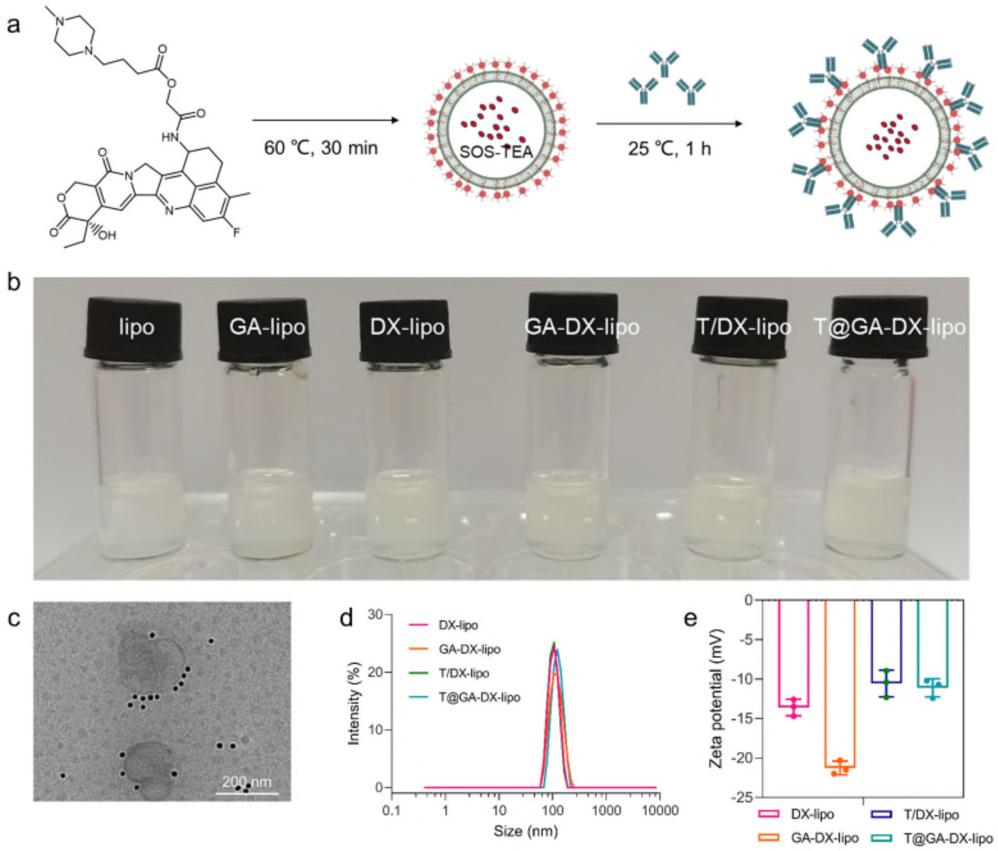

Supplementary Fig.36 The characterization of different T@GA-DX-lipo. a, Illustration of the preparation of T@GA-DX-lipo. **b**, The picture of blank liposome, DXdd-loaded liposome and TRA adsorbed DXdd-loaded liposome. **c,** Immunogold labelling showing the binding of pre-adsorbed TRA antibodies to secondary antibodies on T@GA-DX-lipo. **d**, Size and **e**, zeta potential of DX-lipo, GA-DX-lipo, T/DX-lipo and T@GA-DX-lipo. The mean ± s.d. is displayed from three parallel experiments (n = 3).

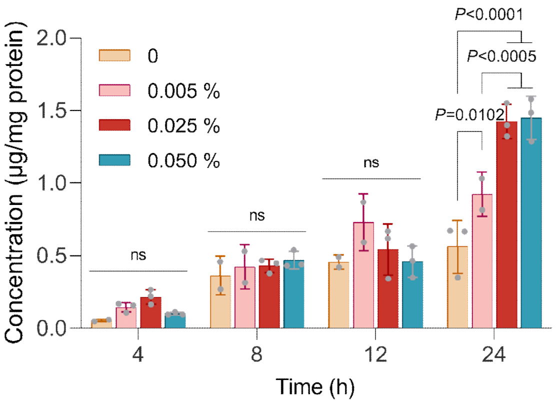

Supplementary Fig.37 The amount of adsorbed TRA on GA-DX-lipo influence the uptake behavior of SKOV3. Incubation of SKOV3 cells with T@GA-DX-lipid (DXdd 5 μg/ml) for 4, 8, 12, 24 h at 37 °C in the presence of 10% FBS. The concentration of uptake DXdd is shown as mean ± s.d. of three independent experiments (n = 3). Statistical significance was analyzed by one-way ANOVA and Tukey's multiple comparisons test.

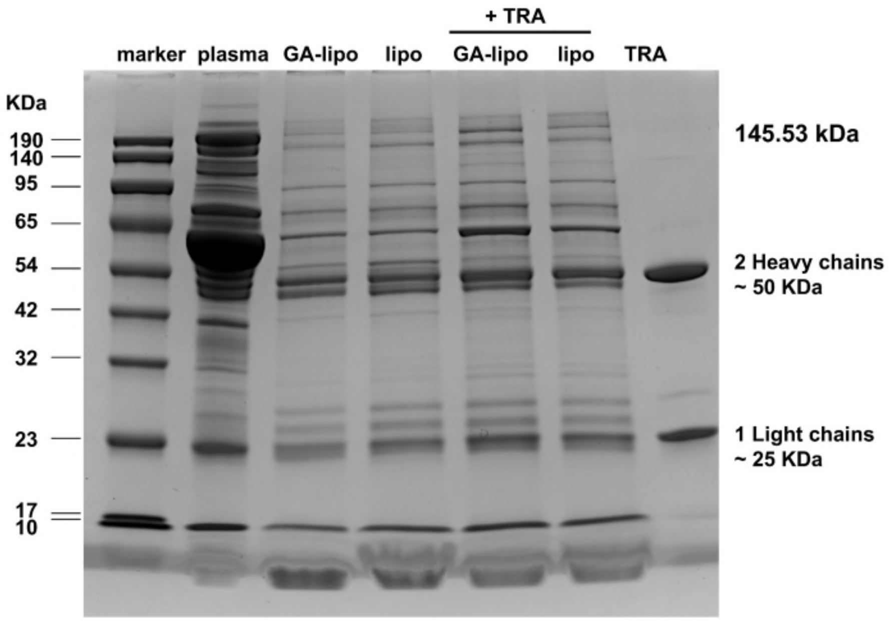

Supplementary Fig.38 Adsorbed TRA remained on the surface of GA-lipo during the formation of protein corona. Liposomes were incubated with 50% mice plasma for 2 h at 37 ℃, isolated via centrifugation and washed three times to remove unbound proteins. Hard corona proteins were desorbed from the particles using 2% SDS, separated by SDS-PAGE and stained with Evans Blue. Experiments were repeated three times.

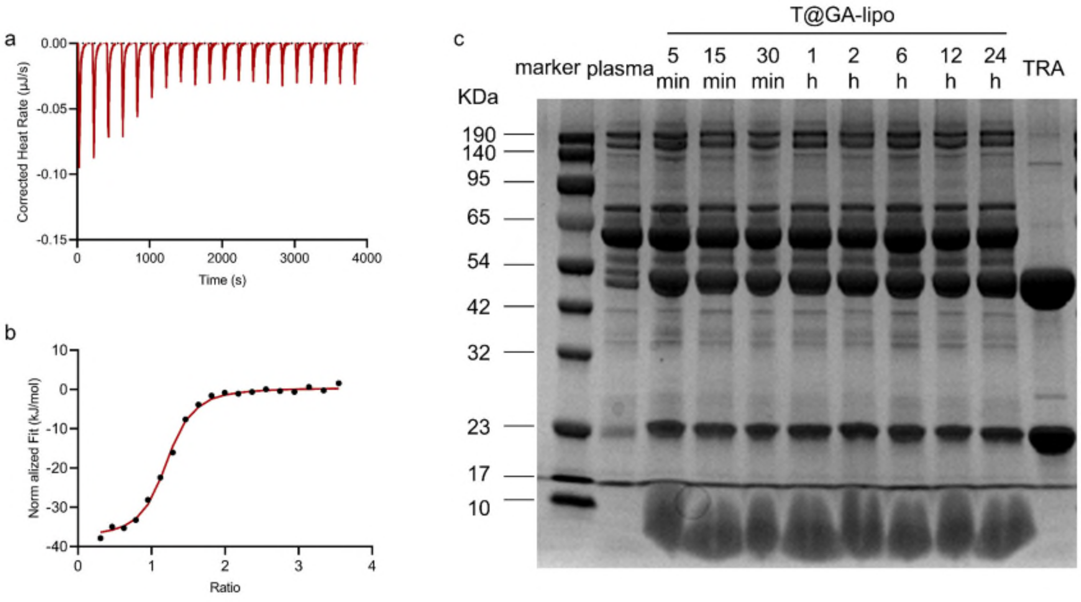

Supplementary Fig.39 Characterization of protein binding and corona formation on T@GA-lipo. a, Isothermal titration calorimetry (ITC) thermogram and b, scatter plot showing the interaction between trastuzumab and GA-lipo. c, Time-dependent formation of the protein corona on T@GA-lipo nanoparticles in mouse plasma. After incubation with plasma for various time points (5 min to 24 h), protein-bound nanoparticles were isolated and analyzed by SDS–PAGE. TRA: free trastuzumab as control. Experiments were repeated three times.

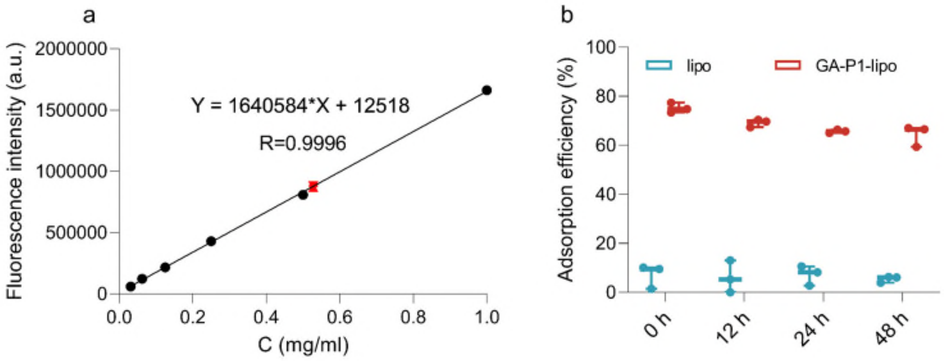

Supplementary Fig.40 Adsorbed TRA on GA-lipo stable in the plasma. a, the standard curve of TRA-FITC. **b,** Liposomes were incubated with 50% mice plasma at 37 °C for 2 h, centrifugation and measured the fluorescence intensity of FITC in the supernatant. The mean ± s.d. is displayed from three parallel experiments (*n* = 3).

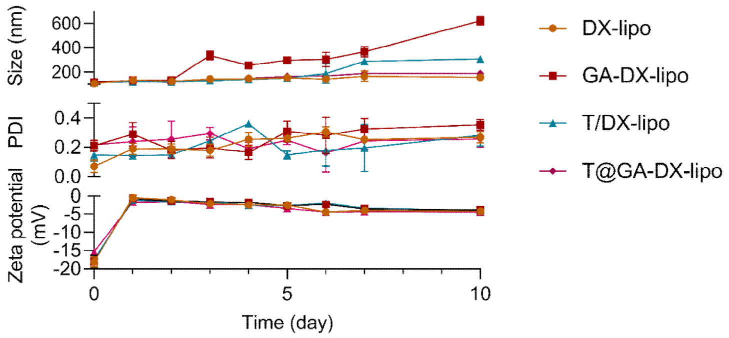

Supplementary Fig.41 Plasma stability of DXdd-loaded formulations. Size, PDI and zeta potential of DX-lipo, GA-DX-lipo, T/DX-lipo and T@GA-DX-lipo in mice plasma at 37 °C for 10 days (mean ± s.d., *n* = 3 independent experiments).

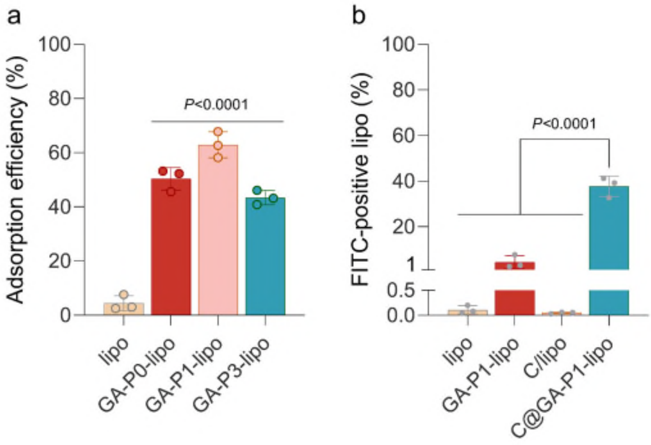

Supplementary Fig.42 Absorption of cetuximab on GA-lipo. a, The influence of the type of GA-Chol on the absorption efficiency of cetuximab. **b**, Detection of Cet with fluorescently (FITC) labelled by flow cytometry. Data are shown as the amount of FITC-positive LPs in percentage and are the mean ± s.d. of three independent experiments (*n* = 3). Statistical significance was analyzed by one-way ANOVA and Tukey's multiple comparisons test.

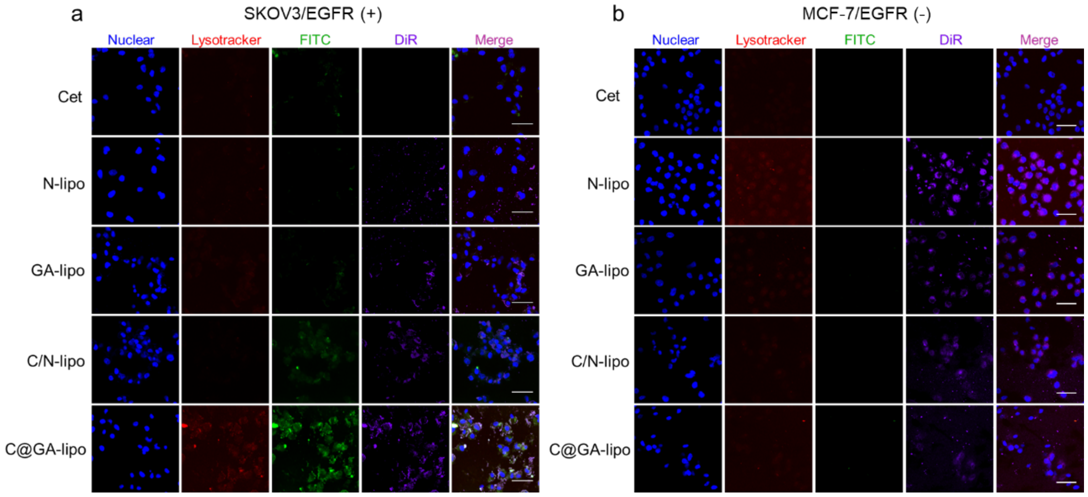

Supplementary Fig.43 Lysosome colocalization experiments. a, Adsorption of Cet to GA-lipo improves uptake on EGFR positive SKOV3 cells. Confocal analysis. After incubation of SKOV3 with different liposomes for 8 h, SKOV3 were stained with Lyso Tracker Red to lable Lysosome. Fluorescence signal of liposome was displayed with DiR. Cet was labeled with FITC. Scale bar: 50 μm. **b**, Adsorption of Cet to GA-lipo have no influence on uptake on EGFR negative MCF-7 cells. Confocal analysis. After incubation of MCF-7 with different liposomes for 8 h, MCF-7 were stained with Lyso Tracker Red to lable Lysosome. Experiments were repeated three times.

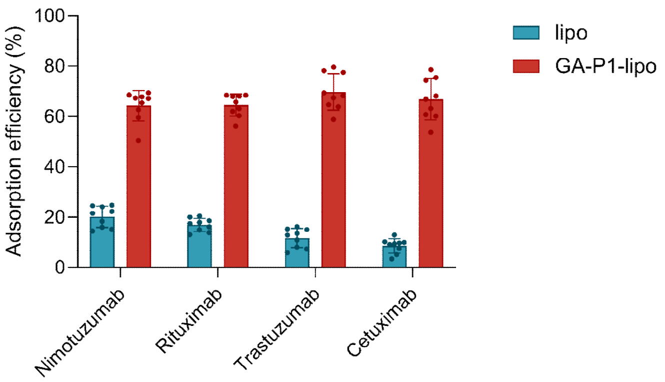

Supplementary Fig.44 The adsorption efficiency of different antibodies. The mean ± s.d. is displayed from nine parallel experiments (n = 9 technical replicates).

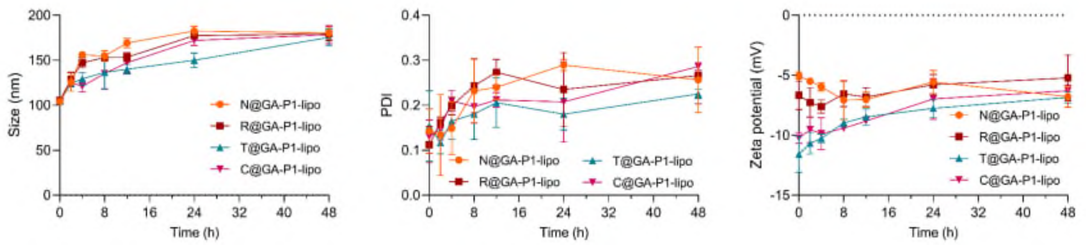

Supplementary Fig.45 Serum stability of antibody-decorated GA-P1-lipo. Time-dependent (0–48 h, 37 °C, 10% FBS) changes in size, PDI and ζ-potential for N@, R@, T@ and C@GA-P1-lipo (mean ± s.d., n = 3 technical replicates).

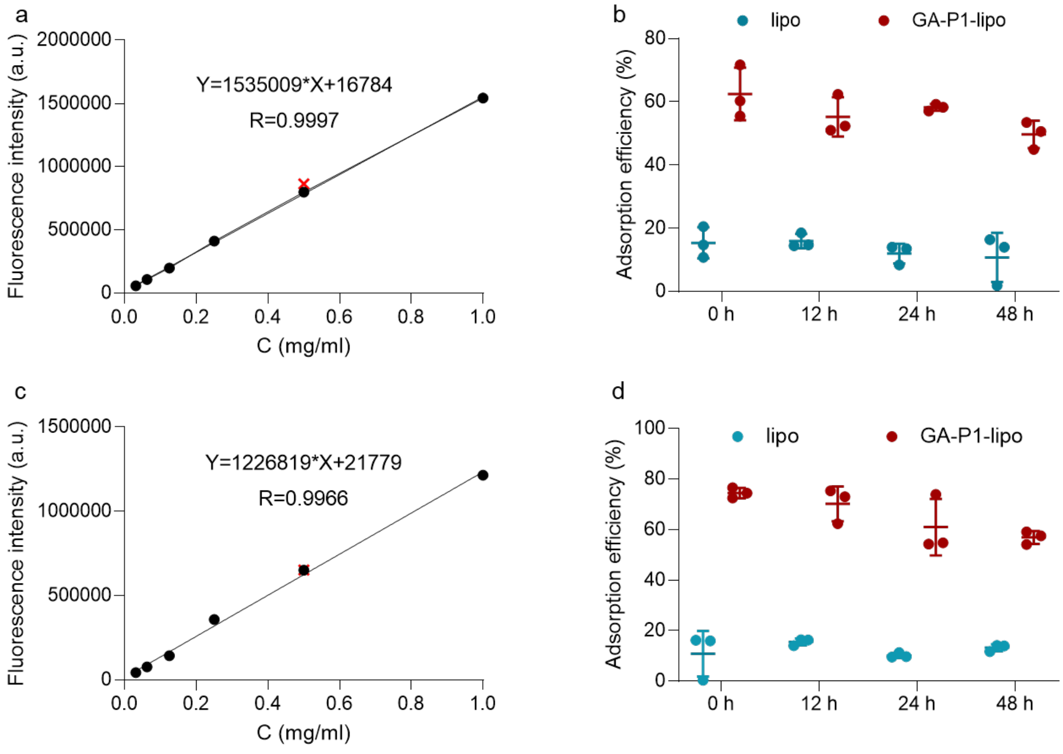

Supplementary Fig.46 Plasma retention of adsorbed antibodies. (a, c) Calibration curves for FITC-labelled nimotuzumab and rituximab. (b, d) Fluorescence remaining on GA-P1-lipo after 48 h in 50% mouse plasma (mean ± s.d., n = 3 technical replicates).

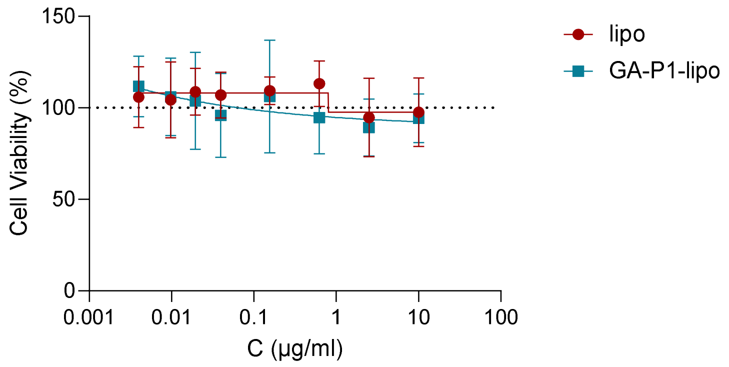

Supplementary Fig.47 Cell viability treated with various concentrations of galloylated and non-galloylated blank liposomes in SKOV3 cells after 48 h. (n = 5 biologically independent samples, mean value ± s.d.).

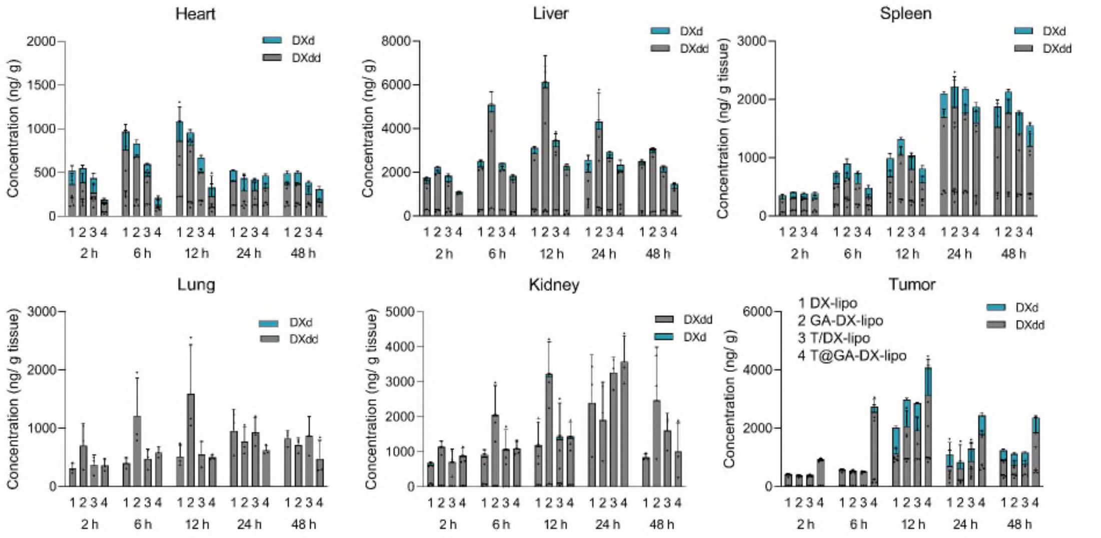

Supplementary Fig.48 In vivo biodistribution of the 4T1 tumor-bearing mice at 2, 6, 12, 24 and 48 h after i.v. administration to mice with DXdd-loaded liposomes at a DXdd equivalent dose of 5 mg/kg (n = 3 biologically independent samples, mean ± s.d.).

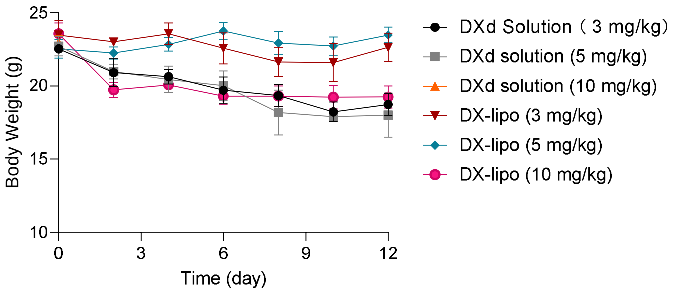

Supplementary Fig.49 Body-weight monitoring after administration of different formulations. BALB/c-nu mice were intravenously injected with free DXd or DXdd-loaded liposomes at doses of 3, 5, or 10 mg/kg, and body weight was monitored over time (n = 3 biologically independent samples, mean ± s.d.).

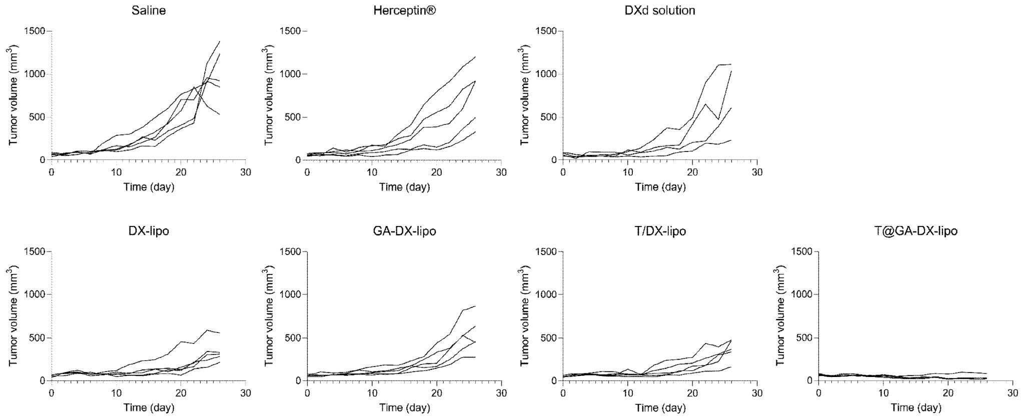

Supplementary Fig.50 Treatment efficacy of T@GA-DX-lipo. Tumor growth curves of mice in each group during treatment cycle.

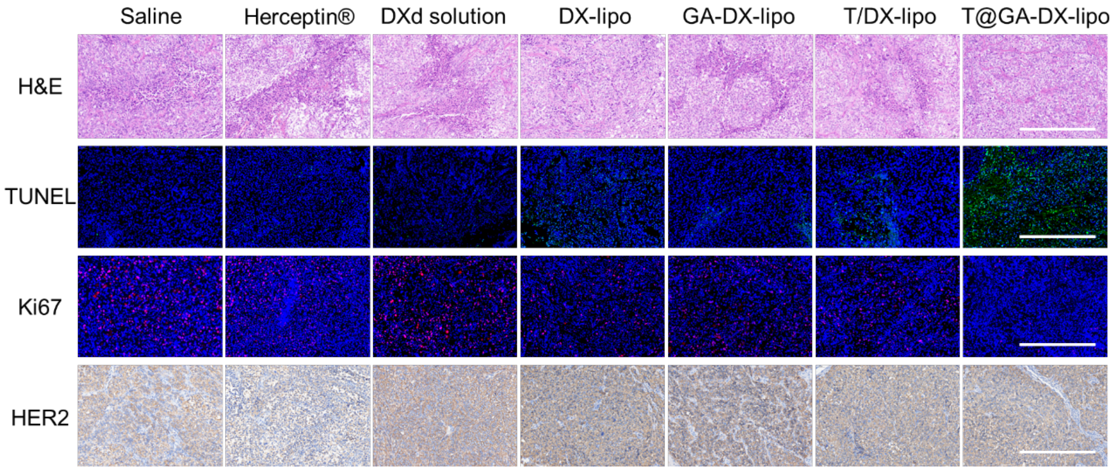

Supplementary Fig.51 Histologic sections and HER2 expression of tumor treatment with DXdd loaded liposomes in vivo. SKOV3 tumors obtained from each group at the end of treatment, Histologic sections of tumors stained with hematoxylin/eosin and Ki67, The HER2 expression of tumors stained with anti-ErbB2 rabbit pAb**.** Scale bar: 500 μm. Experiments were repeated three times.

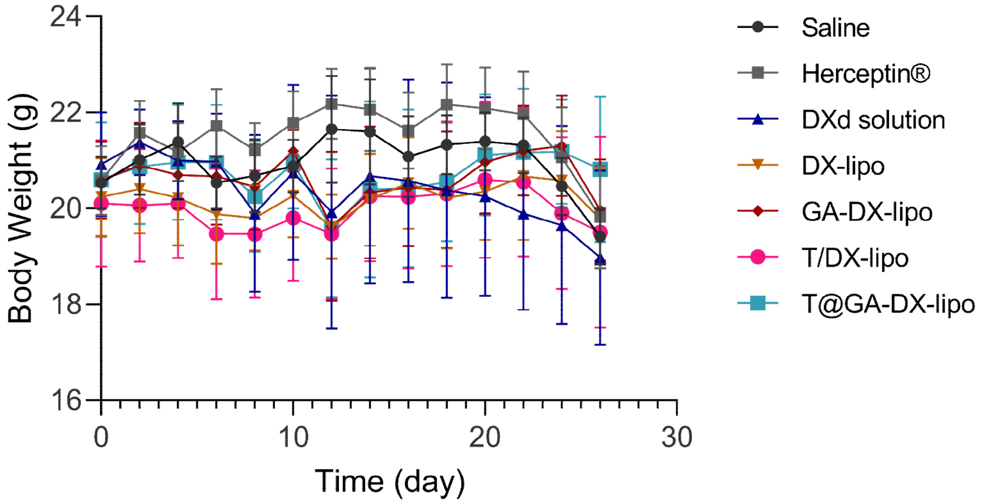

Supplementary Fig.52 Body weight change of mice during treatment cycle. Six biological replicates were measured (n = 6 biologically independent samples, mean values were shown; one-way ANOVA).

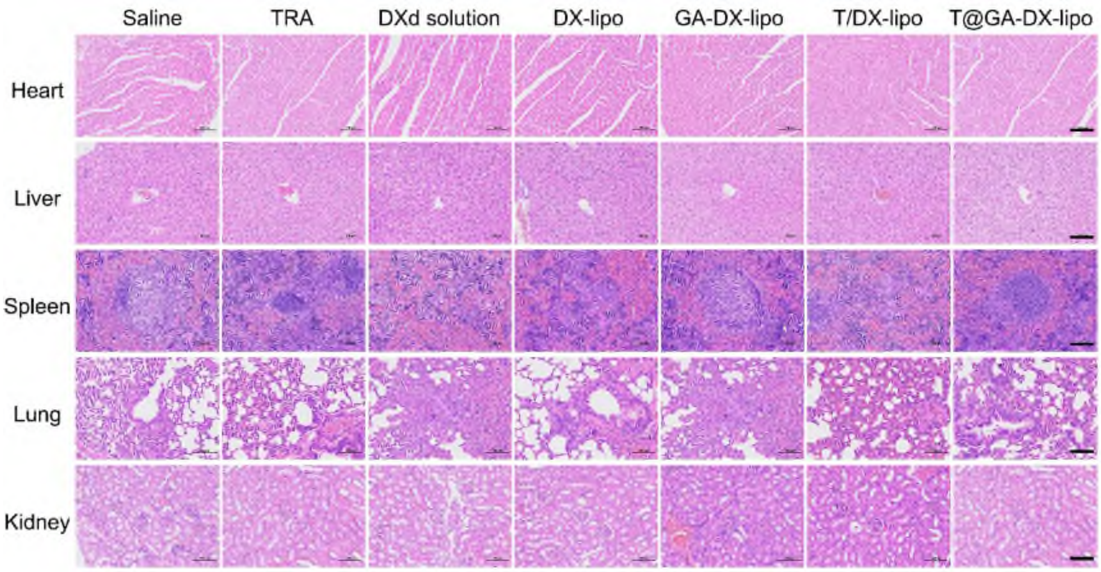

Supplementary Fig.53 Hematoxylin and Eosin staining of major organs in respective groups. Scare bar: 100 μm. Experiments were repeated three times.

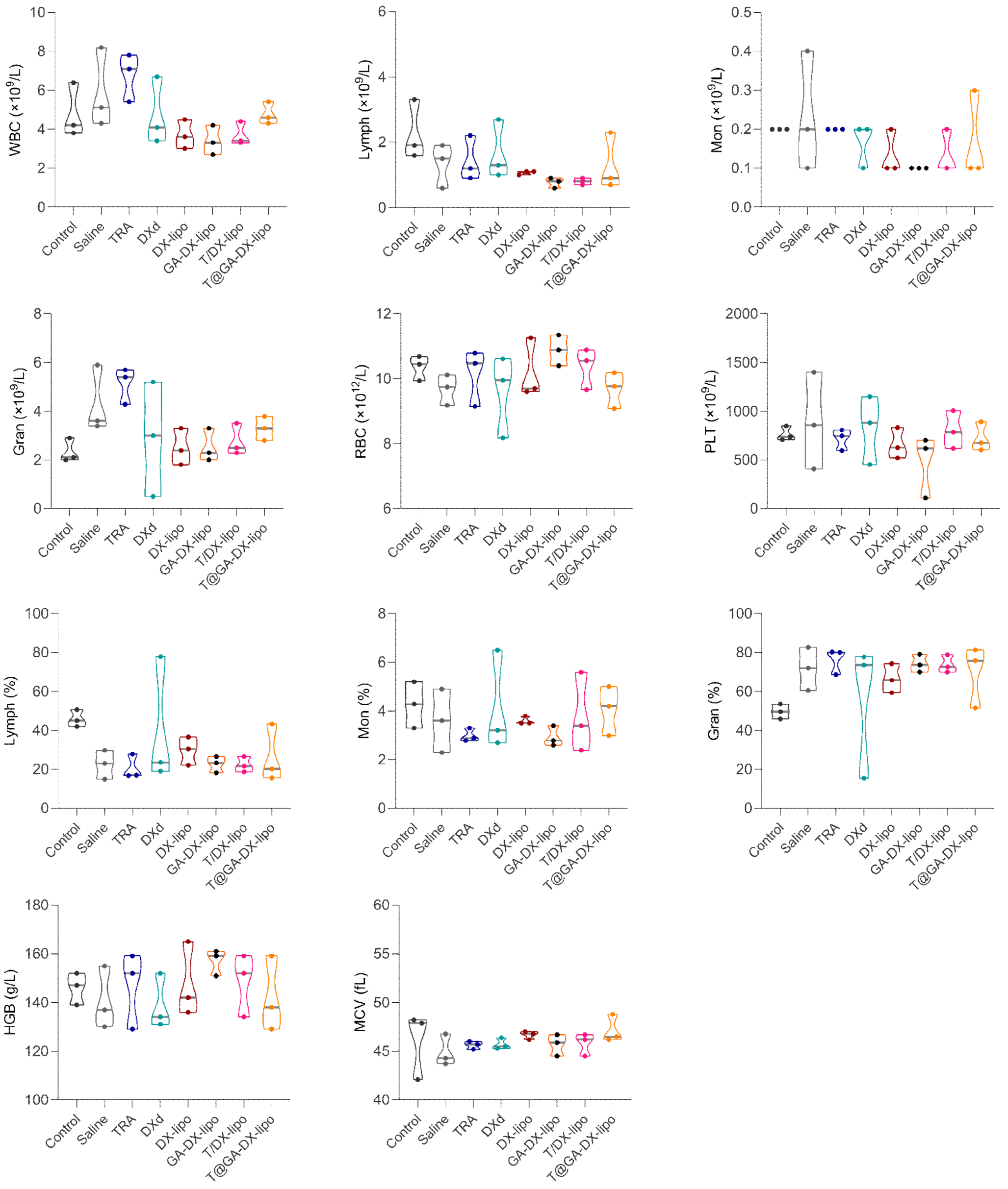

Supplementary Fig.54 Hematological toxicity parameters of mice after treatments. Three biological replicates were measured (*n* = 3).

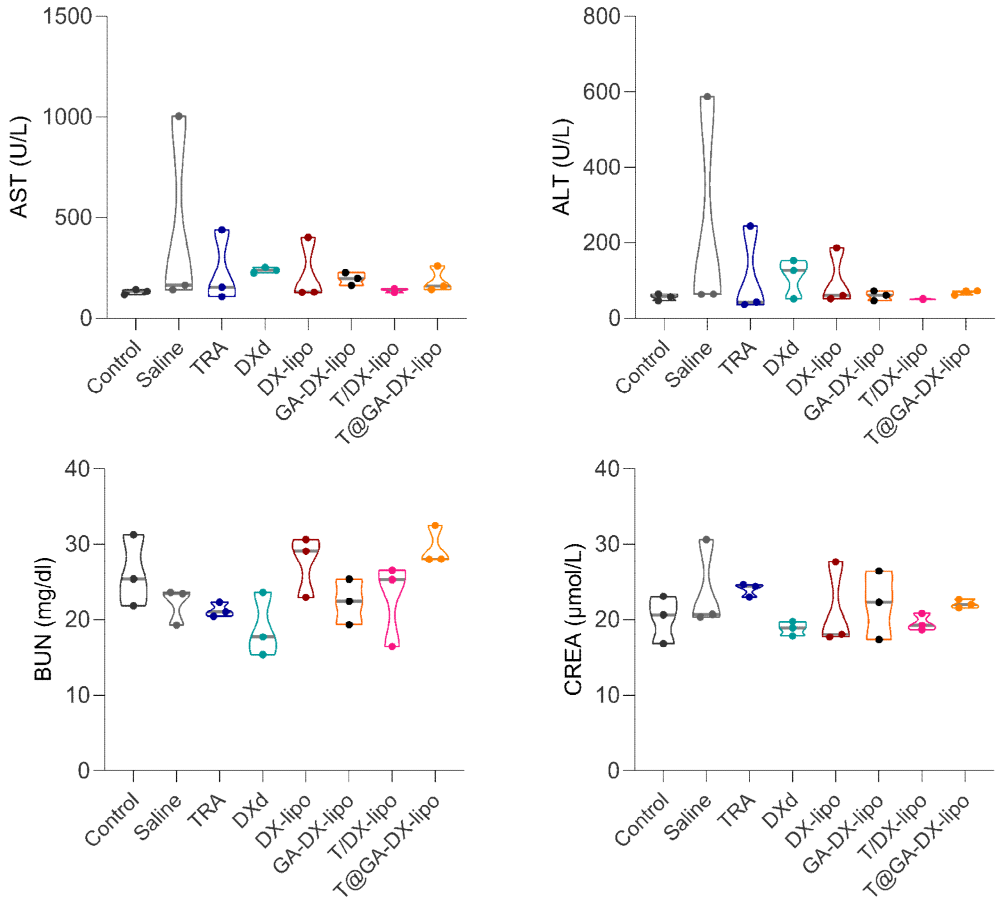

Supplementary Fig.55 Hepatic and renal function parameters of mice after treatments, including ALT, AST, BUN and CREA. Three biological replicates were measured (*n* = 3).

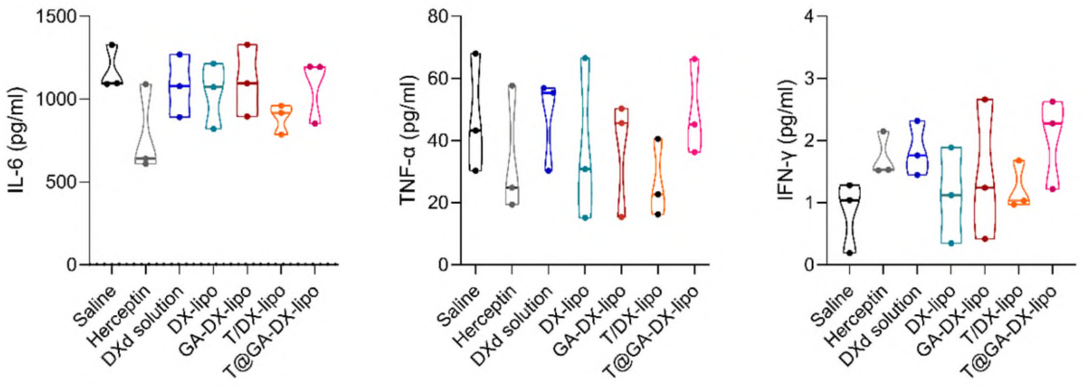

Supplementary Fig.56 Cytokine analysis in serum samples of mice after treatments, including (IL-6, TNF-α, IFN-γ). Three biological replicates were measured (*n* = 3).

Flow cytometry-gating strategy

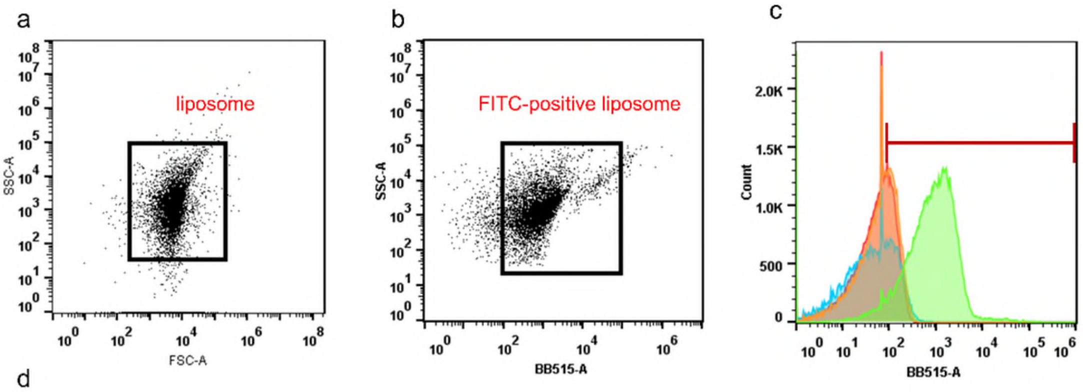

		Median fluorescence intensity MFI (a.u.)	FITC-positive liposomes (%)
Blue	lipo	151	13.7
Orange	GA-lipo	210	16.9
Red	T/lipo	149	11.5
Green	T@GA-lipo	1822	61.4

Supplementary Fig.57 Flow cytometry-gating strategy for the detection of FITC labeled TRA/Cet on liposome surfaces. a, Liposomes were incubated with FITC labeled TRA or Cet. Liposomes were excluded in a forward/side scatter dot plot (FSC vs. SSC) and gate was applied to all samples. b, SSC-A and FL1 channel were scaled logarithmically. c, The medium fluorescence intensity (MFI) was determined via a histogram. The negative control (blank liposome) was set to 0.5% liposome-FITC positive cells as false positive (%). d, Data is shown for one representative measurement from three independent experiments (n = 3).

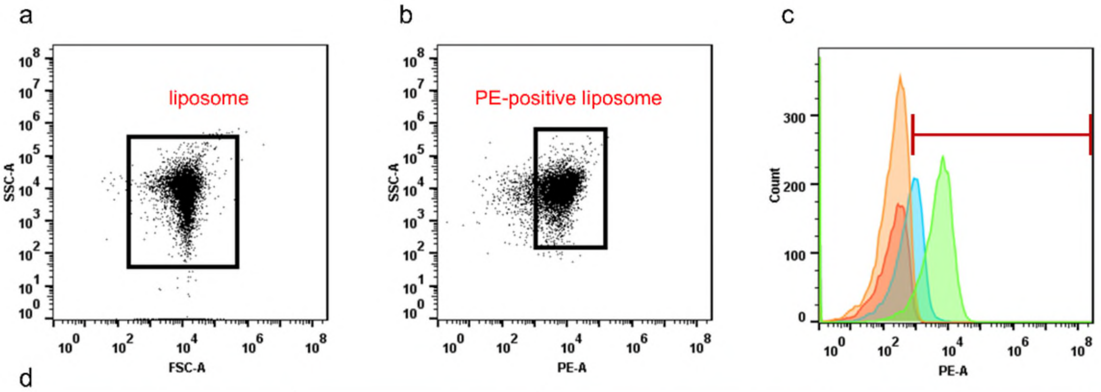

d

		Median fluorescence intensity MFI (a.u.)	PE-positive liposomes (%)
Red	lipo	1094	0.11
Blue	GA-lipo	1722	16.9
Orange	T/lipo	1300	0.18
Green	T@GA-lipo	7587	62.8

Supplementary Fig.58 Flow cytometry-gating strategy for the detection of PE labeled Fab targeted secondary antibody on liposome surfaces. a, Liposomes were incubated with TRA or Cet. After washed three times, PE labeled Fab targeted secondary were incubated with liposomes for 30 min. The stained liposomes were washing for three times further. Then, liposomes were excluded in a forward/side scatter dot plot (FSC vs. SSC) and gate was applied to all samples. b, SSC-A and FL1 channel were scaled logarithmically. c, The medium fluorescence intensity (MFI) was determined via a histogram. The negative control (blank liposome) was set to 0.5% liposome-FITC positive cells as false positive (%). d, Data is shown for one representative measurement from three independent experiments (n = 3).

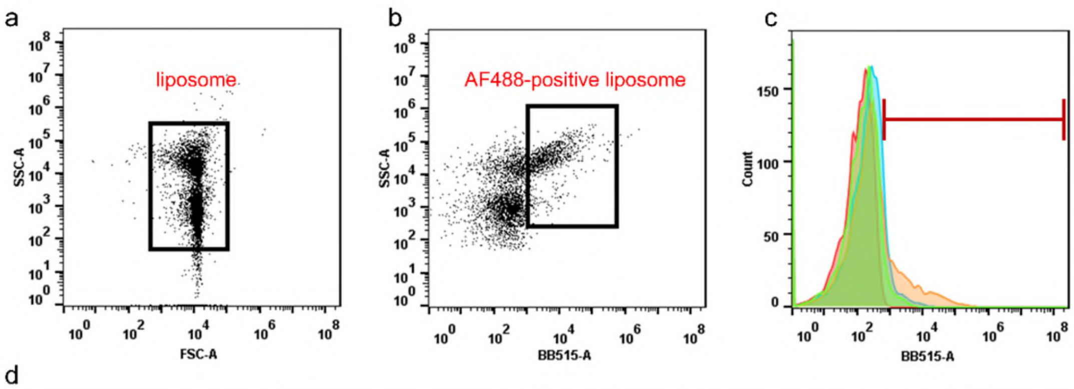

d

		Median fluorescence intensity MFI (a.u.)	AF-488-positive liposomes (%)
Blue	lipo	122	0.53
Red	GA-lipo	109	0.33
Orange	T/lipo	704	9.84
Green	T@GA-lipo	102	0.27

Supplementary Fig.59 Flow cytometry-gating strategy for the detection of AF488 labeled Fab targeted secondary antibody on liposome surfaces. a, Liposomes were incubated with TRA or Cet. After washed three times, AF488 labeled Fab targeted secondary were incubated with liposomes for 30 min. The stained liposomes were washing for three times further. Then, liposomes were excluded in a forward/side scatter dot plot (FSC vs. SSC) and gate was applied to all samples. b, SSC-A and FL1 channel were scaled logarithmically. c, The medium fluorescence intensity (MFI) was determined via a histogram. The negative control (blank liposome) was set to 0.5% liposome-FITC positive cells as false positive (%). d, Data is shown for one representative measurement from three independent experiments (*n* = 3).

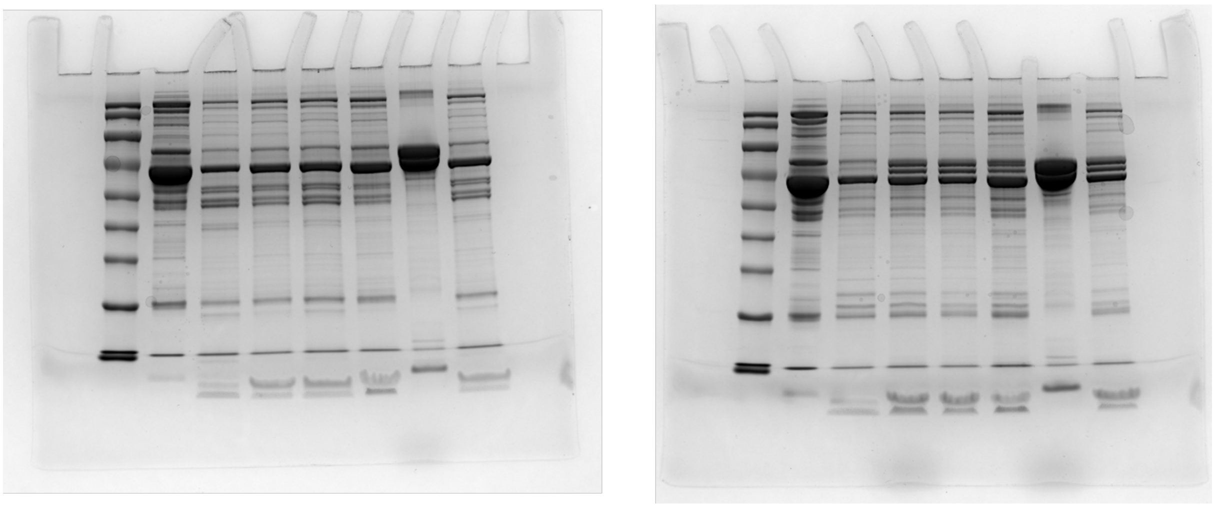

Supplementary Fig.60 The uncropped scans of gels in supplementary Fig. 21.

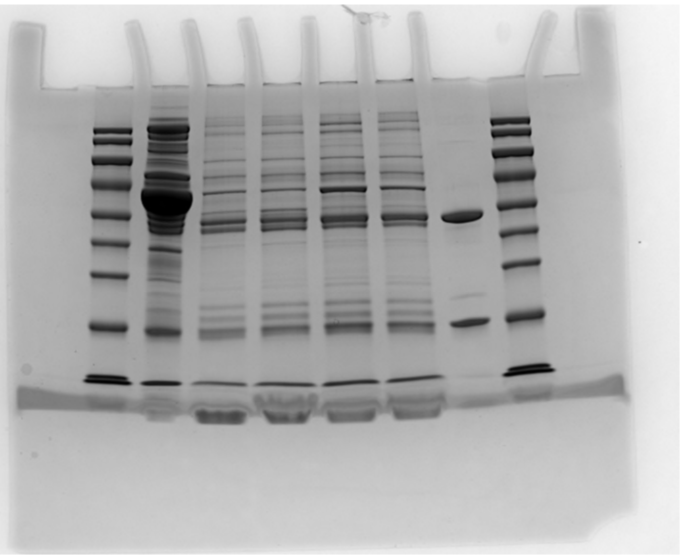

Supplementary Fig.61 The uncropped scans of gels in supplementary Fig. 38.

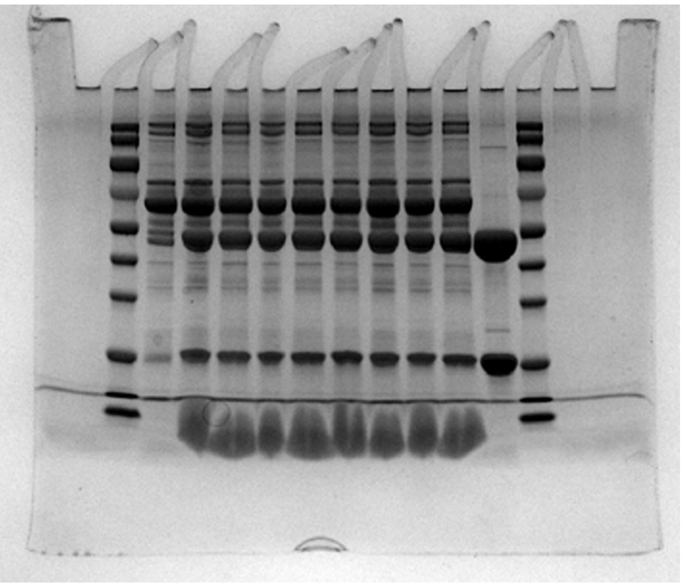

Supplementary Fig.62 The uncropped scans of gels in supplementary Fig. 39c.
