# Supplementary material for: Galloylated liposomes enable targeted drug delivery by overcoming protein corona shielding
Source: Nat Commun. 2025 Aug 25;16:7926. doi: 10.1038/s41467-025-63198-4 (PMC12378351; doi:10.1038/s41467-025-63198-4)
Supplement: Supplementary file 2 — Reporting Summary [file 41467_2025_63198_MOESM2_ESM.pdf]

nature portfolio

Corresponding author(s): Yongjun Wang

Last updated by author(s): Jul 21, 2025

Reporting Summary

Nature Portfolio wishes to improve the reproducibility of the work that we publish. This form provides structure for consistency and transparency in reporting. For further information on Nature Portfolio policies, see our Editorial Policies and the Editorial Policy Checklist.

Statistics

For all statistical analyses, confirm that the following items are present in the figure legend, table legend, main text, or Methods section.

n/a	Confirmed	
☐	☒	The exact sample size (*n*) for each experimental group/condition, given as a discrete number and unit of measurement
☐	☒	A statement on whether measurements were taken from distinct samples or whether the same sample was measured repeatedly
☐	☒	The statistical test(s) used AND whether they are one- or two-sided<br>*Only common tests should be described solely by name; describe more complex techniques in the Methods section.*
☐	☒	A description of all covariates tested
☐	☒	A description of any assumptions or corrections, such as tests of normality and adjustment for multiple comparisons
☐	☒	A full description of the statistical parameters including central tendency (e.g. means) or other basic estimates (e.g. regression coefficient) AND variation (e.g. standard deviation) or associated estimates of uncertainty (e.g. confidence intervals)
☐	☒	For null hypothesis testing, the test statistic (e.g. *F*, *t*, *r*) with confidence intervals, effect sizes, degrees of freedom and *P* value noted<br>*Give P values as exact values whenever suitable.*
☒	☐	For Bayesian analysis, information on the choice of priors and Markov chain Monte Carlo settings
☒	☐	For hierarchical and complex designs, identification of the appropriate level for tests and full reporting of outcomes
☒	☐	Estimates of effect sizes (e.g. Cohen's *d*, Pearson's *r*), indicating how they were calculated

Our web collection on statistics for biologists contains articles on many of the points above.

Software and code

Policy information about availability of computer code

Data collection	HPLC: Empower 3; Malvern: Zetasizer Software 7.01; Confocal laser scanning microscopy: NIS 4.13; Flow Cytometer: BD CellQuest Pro
Data analysis	MestReNova 15.0.0; GraphPad prism 9.0.0; Flowjo v10; Origin 10.1.0.178

For manuscripts utilizing custom algorithms or software that are central to the research but not yet described in published literature, software must be made available to editors and reviewers. We strongly encourage code deposition in a community repository (e.g. GitHub). See the Nature Portfolio guidelines for submitting code & software for further information.

Data

Policy information about availability of data

All manuscripts must include a data availability statement. This statement should provide the following information, where applicable:

- Accession codes, unique identifiers, or web links for publicly available datasets
- A description of any restrictions on data availability
- For clinical datasets or third party data, please ensure that the statement adheres to our policy

All data supporting the findings of this study are available within the paper and its Supplementary Information.

Research involving human participants, their data, or biological material

Policy information about studies with human participants or human data. See also policy information about sex, gender (identity/presentation), and sexual orientation and race, ethnicity and racism.

Reporting on sex and gender	not applicable
Reporting on race, ethnicity, or other socially relevant groupings	not applicable
Population characteristics	not applicable
Recruitment	not applicable
Ethics oversight	not applicable

Note that full information on the approval of the study protocol must also be provided in the manuscript.

Field-specific reporting

Please select the one below that is the best fit for your research. If you are not sure, read the appropriate sections before making your selection.

[x] Life sciences [ ] Behavioural & social sciences [ ] Ecological, evolutionary & environmental sciences

For a reference copy of the document with all sections, see nature.com/documents/nr-reporting-summary-flat.pdf

Life sciences study design

All studies must disclose on these points even when the disclosure is negative.

Sample size	No statistical methods were used to predetermine sample sizes, but sample sizes used in this study are comparable to those reported in previous publications (see References 16, 17, 21).
Data exclusions	No data were excluded from the analyses.
Replication	We confirmed that all repeated attempts were successful. The number of experiment repetitions is provided in the Figure Legends.
Randomization	Samples were randomly allocated into experimental groups.
Blinding	Experiments were blinded to treatment group during data collection and image or electrophysiological analysis

Reporting for specific materials, systems and methods

We require information from authors about some types of materials, experimental systems and methods used in many studies. Here, indicate whether each material, system or method listed is relevant to your study. If you are not sure if a list item applies to your research, read the appropriate section before selecting a response.

Materials & experimental systems

n/a	Involved in the study	
[ ]	[x]	Antibodies
[ ]	[x]	Eukaryotic cell lines
[x]	[ ]	Palaeontology and archaeology
[ ]	[x]	Animals and other organisms
[x]	[ ]	Clinical data
[x]	[ ]	Dual use research of concern
[x]	[ ]	Plants

Methods

n/a	Involved in the study	
[x]	[ ]	ChIP-seq
[ ]	[x]	Flow cytometry
[x]	[ ]	MRI-based neuroimaging

Antibodies

Antibodies used	12 nm Colloidal Gold AffiniPure™ Goat Anti-Human IgG (H+L) (Jackson ImmunoResearch Inc, AB_2337748). The dilution was 1:20.<br>Goat Anti-Human IgG Fc (DyLight® 488) (Abcam, ab97003). The dilution was 1:50.<br>F(ab')$_2$ Fragment Goat Anti-Mouse IgG H&L, PE conjugated (BIOSS, bs-60296G-PE). The dilution was 1:50.<br>Anti-HER2 Rabbit pAb (BIOSS, bs-2156R). The dilution was 1:50.
Validation	Antibodies were validated by manufacturers via a combination of SDS-PAGE, staining of multiple endogenous cell types to confirm

specificity, testing of comparable MFI between lots, and titration assays.

Eukaryotic cell lines

Policy information about cell lines and Sex and Gender in Research

Cell line source(s)	4T1 cells (female), MCF7 cells (female) and SKOV3 cells (female) were obtained from Wuhan Pricella Biotechnology Co., Ltd. (Wuhan, China)
Authentication	All cell lines validation were performed by manufacturers using short tandem repeat (STR) markers. (https://www.procell.com.cn/view/1408.html; https://www.procell.com.cn/view/827.html; https://www.procell.com.cn/view/473.html)
Mycoplasma contamination	All cell lines tested negative for mycoplasma contamination.
Commonly misidentified lines (See ICLAC register)	No commonly misidentified cells lines were used in the study.

Animals and other research organisms

Policy information about studies involving animals; ARRIVE guidelines recommended for reporting animal research, and Sex and Gender in Research

Laboratory animals	Sprague-Dawley rats (male, 6-8 weeks), BALB/c mice (female, 6-8 weeks) and BALB/c-nu mice (female, 6-8 weeks) were supplied by the Animal Center of Shenyang Pharmaceutical University (Shenyang, Liaoning, China). The living environment of animals were maintained at a temperature of ~25 °C with a 12 h light/dark cycle, with free access to standard food and water.
Wild animals	N/A
Reporting on sex	Although our study utilized single-sex animal models, we believe that the findings are not limited to a specific sex. In the pharmacokinetic experiments, male rats were employed, and sex did no influence on the pharmacokinetic outcomes. Given the nature of the selected model, which focused on breast cancer and HER2(+) ovarian cancer, female animals were chosen for the pharmacodynamic investigations.
Field-collected samples	N/A
Ethics oversight	All the animal experiments were conducted according to the Guidelines for the Care and Use of Laboratory Animals approved by the Institutional Animal Ethical Care Committee (IAEC) of Shenyang Pharmaceutical University.

Note that full information on the approval of the study protocol must also be provided in the manuscript.

Plants

Seed stocks	not applicable
Novel plant genotypes	not applicable
Authentication	not applicable

Flow Cytometry

Plots

Confirm that:

- [x] The axis labels state the marker and fluorochrome used (e.g. CD4-FITC).
- [x] The axis scales are clearly visible. Include numbers along axes only for bottom left plot of group (a 'group' is an analysis of identical markers).
- [x] All plots are contour plots with outliers or pseudocolor plots.
- [x] A numerical value for number of cells or percentage (with statistics) is provided.

Methodology

Sample preparation	Details regarding all sample preparation procedures are listed in the manuscript.

Instrument	BD FACSCalibur
Software	BD CellQuest Pro; Flowjo_V10
Cell population abundance	This manuscript used flow cytometry, however, we did not perform physical cell sorting (i.e. FACS) in any of the experiments.
Gating strategy	The gating strategy is described in detail in both the methods section and in the supplementary materials of the manuscript.

☒ Tick this box to confirm that a figure exemplifying the gating strategy is provided in the Supplementary Information.
